# Supplementary material for: Allele-specific transcriptional effects of subclonal copy number alterations enable genotype-phenotype mapping in cancer cells
Source: Nat Commun. 2024 Mar 20;15:2482. doi: 10.1038/s41467-024-46710-0 (PMC10954741; doi:10.1038/s41467-024-46710-0)
Supplement: Supplementary file 1 — Supplementary Information [file 41467_2024_46710_MOESM1_ESM.pdf]

Variable	Distribution	Description
x_{ng}	Multinomial	Gene expression read count
y_{ng}	Deterministic f^n	Modeled expected expression
z_n	Categorical	Clone assignment indicator
π_c	Dirichlet	Prior probability of clone assignment
λ_{gc}		Copy number
μ_g	Softplus-Normal	Per-copy expression or CN-independent base expression
k_g	Bernoulli	Copy number dependency indicator
$p(k)_g$	Beta	Prior probability of CN dependency
$\psi_n \cdot w_g^T$		Structured noise to avoid overfitting
t_{ns}		Total read count at SNPs in scRNA-seq
r_{ns}	Binomial	Reference allele count at SNPs in scRNA-seq
f_{ns}	Deterministic f^n	Reference allele frequency at SNPs in scRNA-seq
b_{sc}		B allele frequency at SNPs in scDNA-seq
a_s	Bernoulli	Allele assignment indicator
$p(a)_s$	Beta	Prior probability for allele assignment indicator

Fig. S1. Random variables and data in TreeAlign. Descriptions and prior distributions of random variables and data in TreeAlign model.

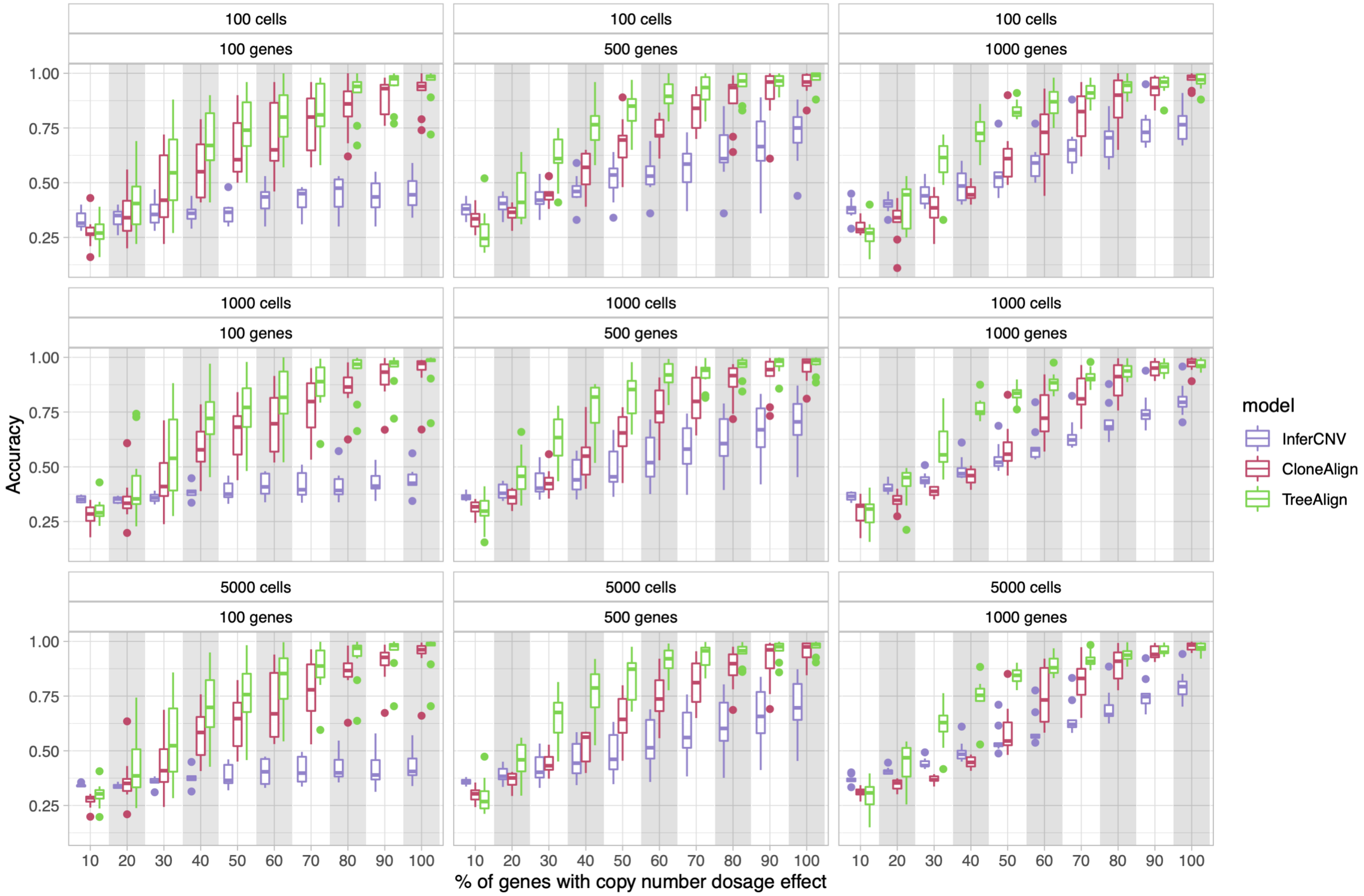

Fig. S2. Clone assignment accuracy of TreeAlign with clone label input in simulated datasets. Accuracy of clone assignment for TreeAlign, CloneAlign and InferCNV in simulated scRNA datasets as a function of varying proportions of genes with CN dosage effects. Panels represent datasets with different numbers of cells and genes.

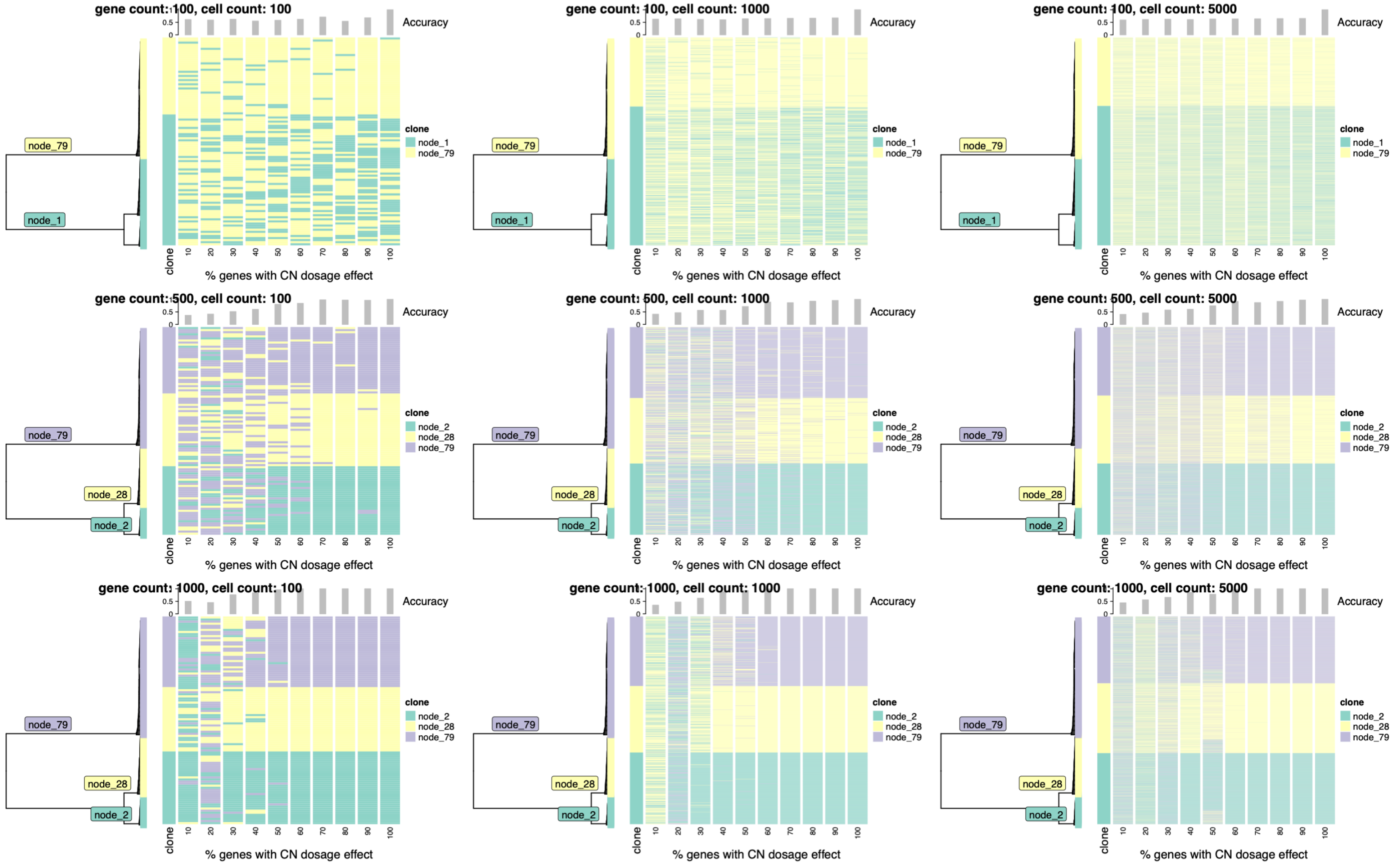

Fig. S3. Clone assignment accuracy of TreeAlign with phylogenetic tree input in simulated datasets. Phylogenetic trees (left) constructed with scDNA-data from SPECTRUM-OV-081 along with Heat maps (right) showing clone assignment of simulated datasets by TreeAlign.

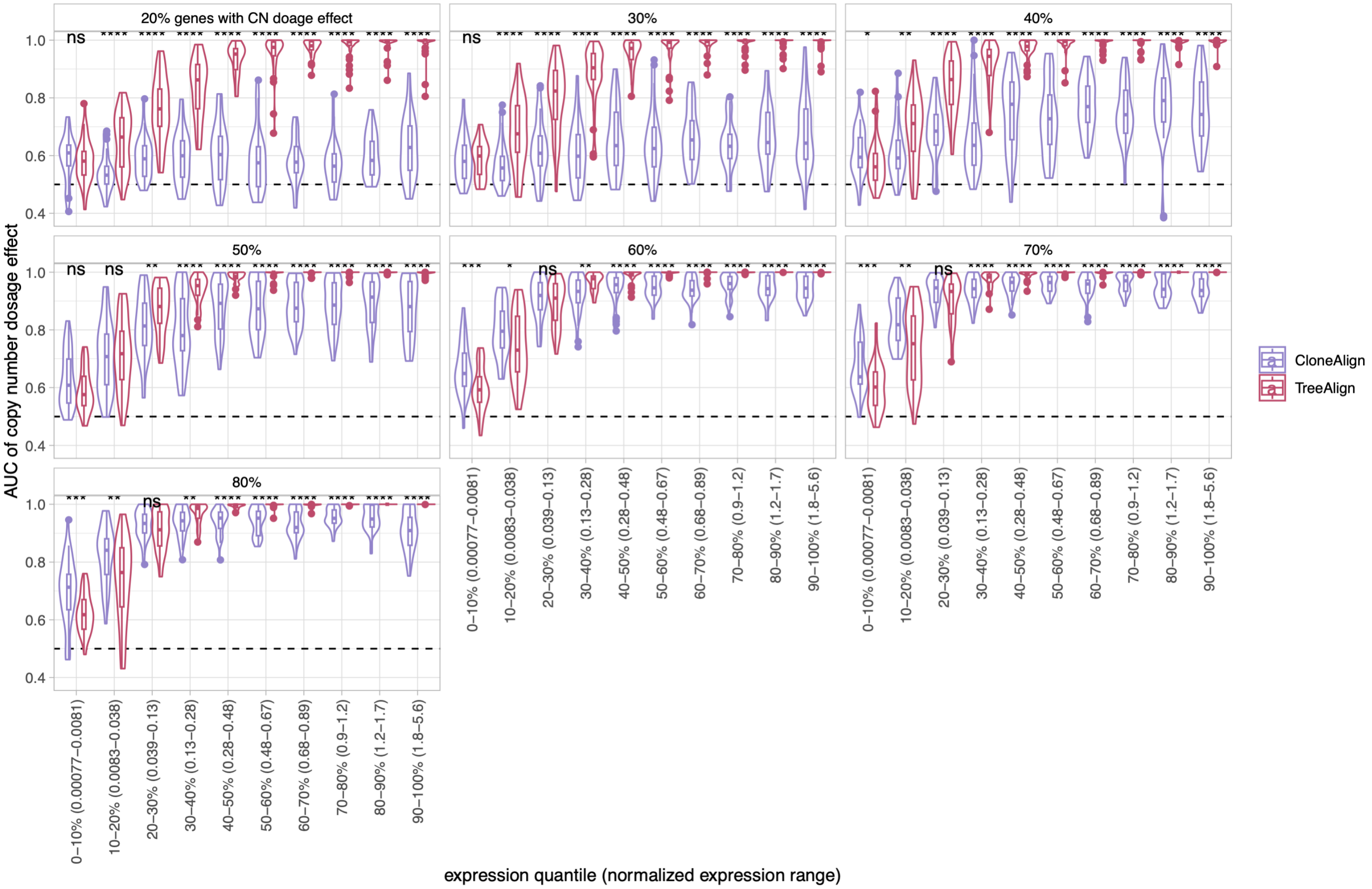

Fig. S4. Dosage effect prediction of TreeAlign in simulated datasets. AUC of CN dosage effect $p(k)$ predicted by CloneAlign and TreeAlign as a function of gene expression level. Genes were assigned to 10 bins based on expression level. Ranges of normalized expression for each bin were shown in brackets. Panels represent simulated datasets with varying gene dosage effect frequencies ($*P < 0.05$, $**P < 0.01$, $***P < 0.001$, $****P < 0.0001$, Two-sided Wilcoxon signed-rank test). For the box plots, box limits extend from the 25th to 75th percentile, while the middle line represents the median. Whiskers extend to the largest value no further than 1.5 times the inter-quartile range (IQR) from each box hinge. Points beyond the whiskers are outliers.

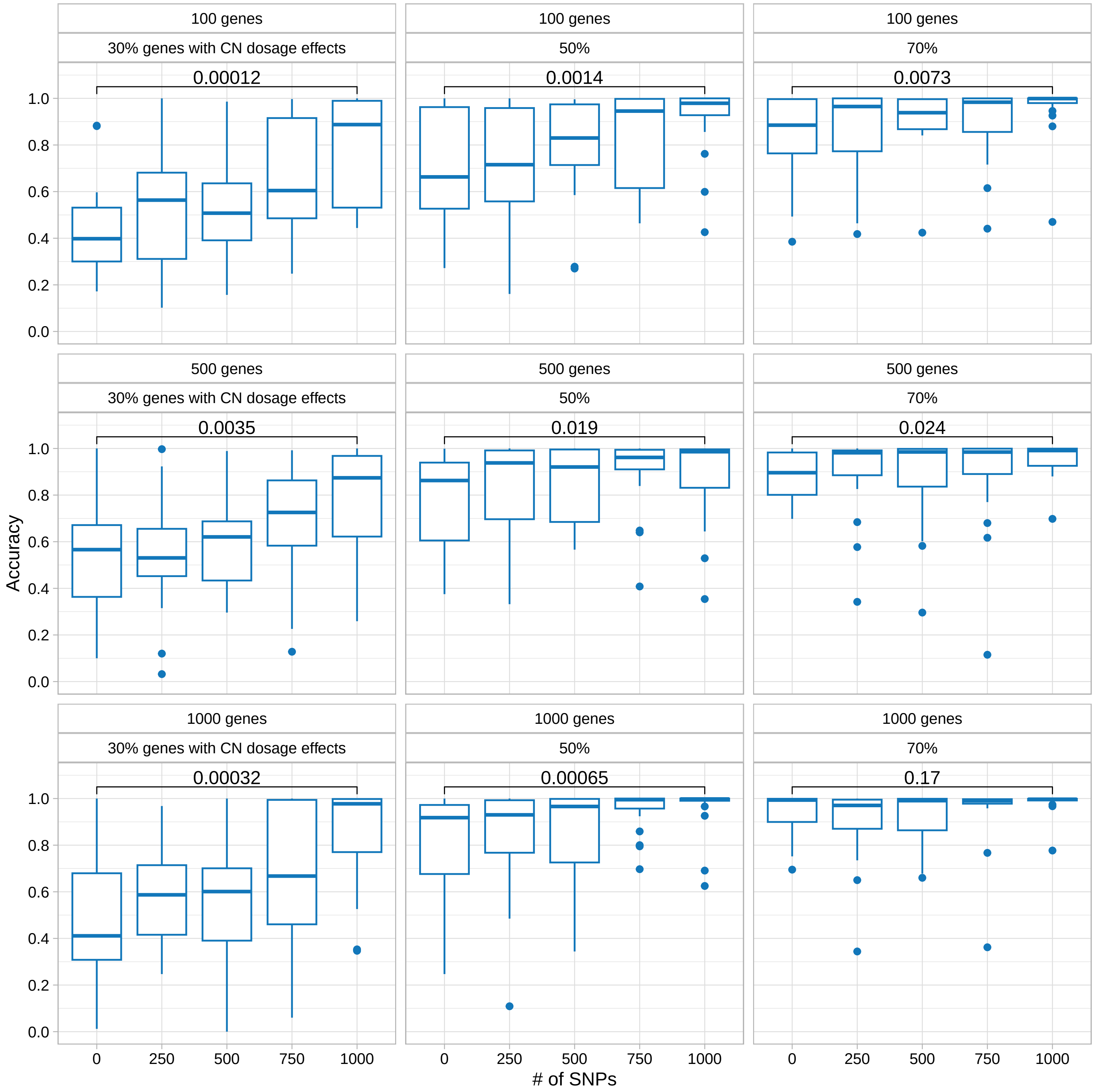

Fig. S5. Clone assignment accuracy with simulated allelic data. Accuracy of clone assignment for the integrated model of TreeAlign on simulated scRNA datasets as a function of varying numbers of heterozygous SNPs in input. Panels represent datasets with different numbers of genes and proportions of genes with CN dosage effects (Two-sided Wilcoxon signed-rank test). For the box plots, box limits extend from the 25th to 75th percentile, while the middle line represents the median. Whiskers extend to the largest value no further than 1.5 times the inter-quartile range (IQR) from each box hinge. Points beyond the whiskers are outliers.

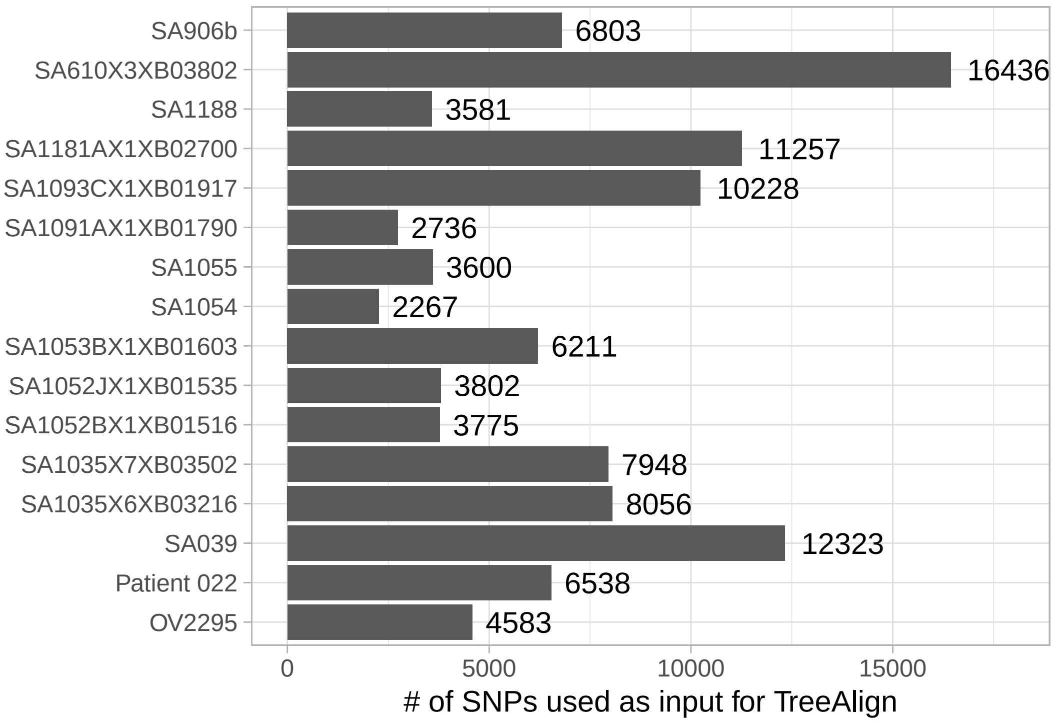

Fig. S6. Number of heterozygous SNPs used as input for TreeAlign in samples from Funnell et al. and patient 022.
.

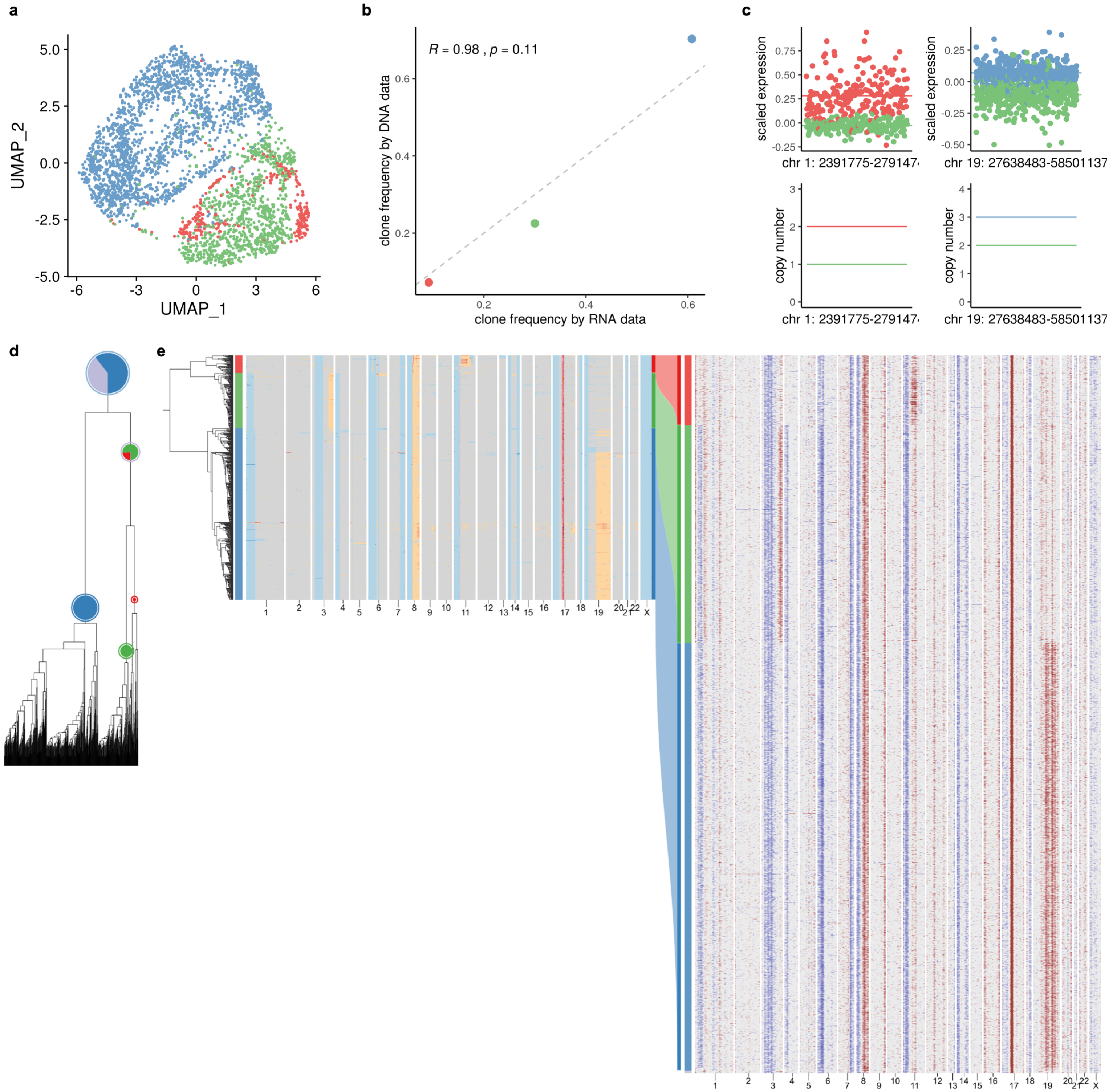

Fig. S7. TreeAlign assigns expression profiles of NCI-N87 to phylogeny. a, UMAP plot of scRNA-data from gastric cell line NCI-N87 colored by clone labels assigned by total CN TreeAlign. **b,** Clone frequencies of NCI-N87 estimated by scRNA-data (x axis) and scDNA-data (y axis). Pearson correlation coefficients (R) and P values for the linear fit (Two-sided Student's t-test) are shown. **c,** Scaled expression and copy number profiles for regions on chromosome 1 and 19 as a function of genes ordered by genomic locations. d, Phylogenetic tree constructed with scDNA-data. **e,** Phylogenetic tree constructed with scDNA-data along with pie charts showing how TreeAlign assigns cell expression profiles to subtrees recursively. The pie charts are colored by the proportions of cell expression profiles assigned to downstream subtrees. The outer ring color of the pie charts indicates the current subtree. Heat maps of copy number profiles from scDNA (left) and InferCNV corrected expression profiles from scRNA (right). The Sankey chart in the middle shows clone assignment from expression profiles to copy number based clones by total CN TreeAlign.

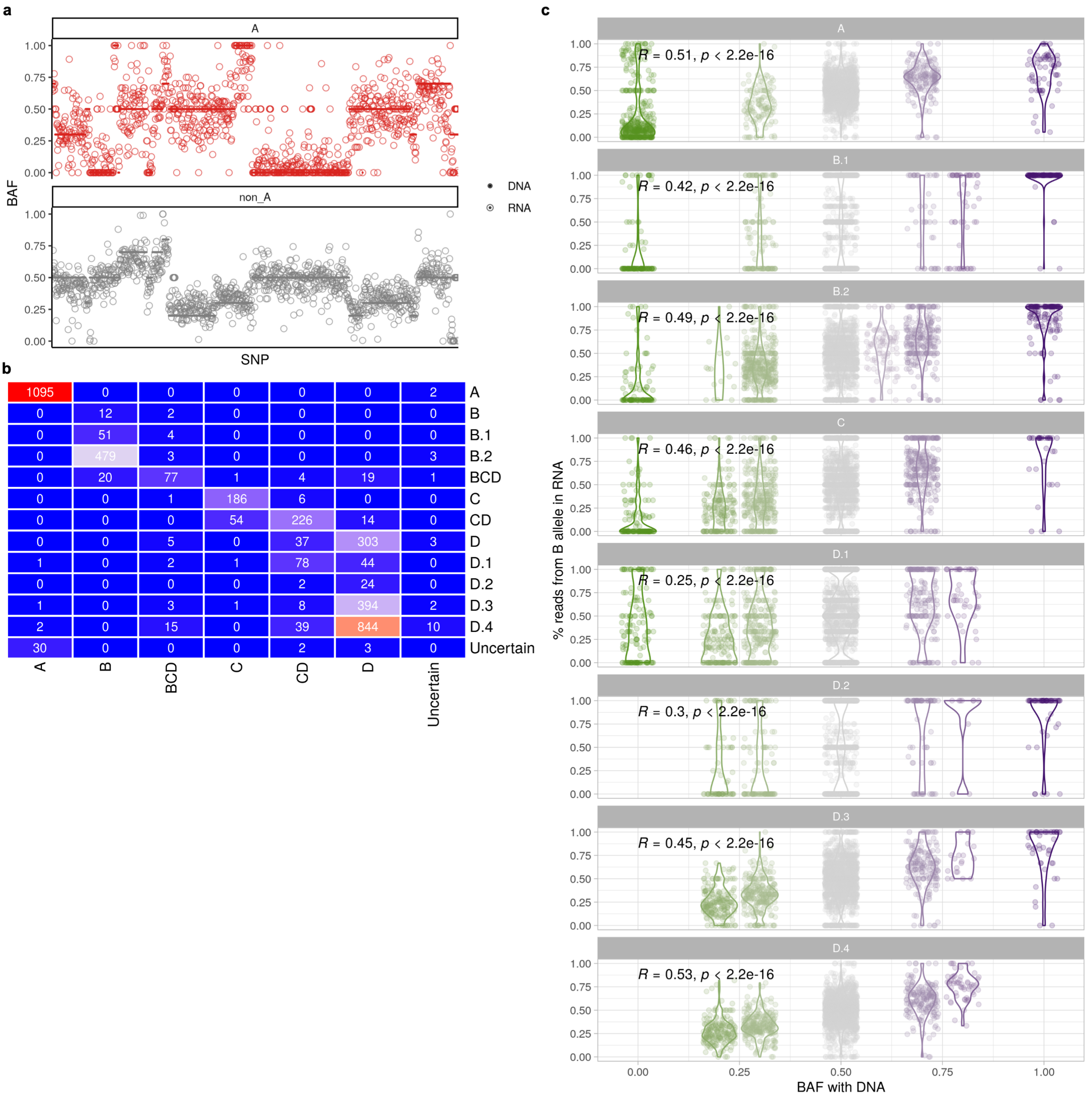

Fig. S8. Allele-specific information contributes to clone assignment. a, BAF of heterozygous SNPs estimated from scRNA-data and scDNA-data for clone A and other clones (clone B - C) in patient 022 (ordered by gene location along chromosome). **b,** Confusion matrix comparing clone assignment between total CN TreeAlign and integrated TreeAlign for patient 022. **c,** Correlation between proportions of reads from B allele in scRNA and BAF estimated from scDNA in patient 022 subclones. Pearson correlation coefficients (R) and P values for the linear fit (Two-sided Student's t-test) are shown..

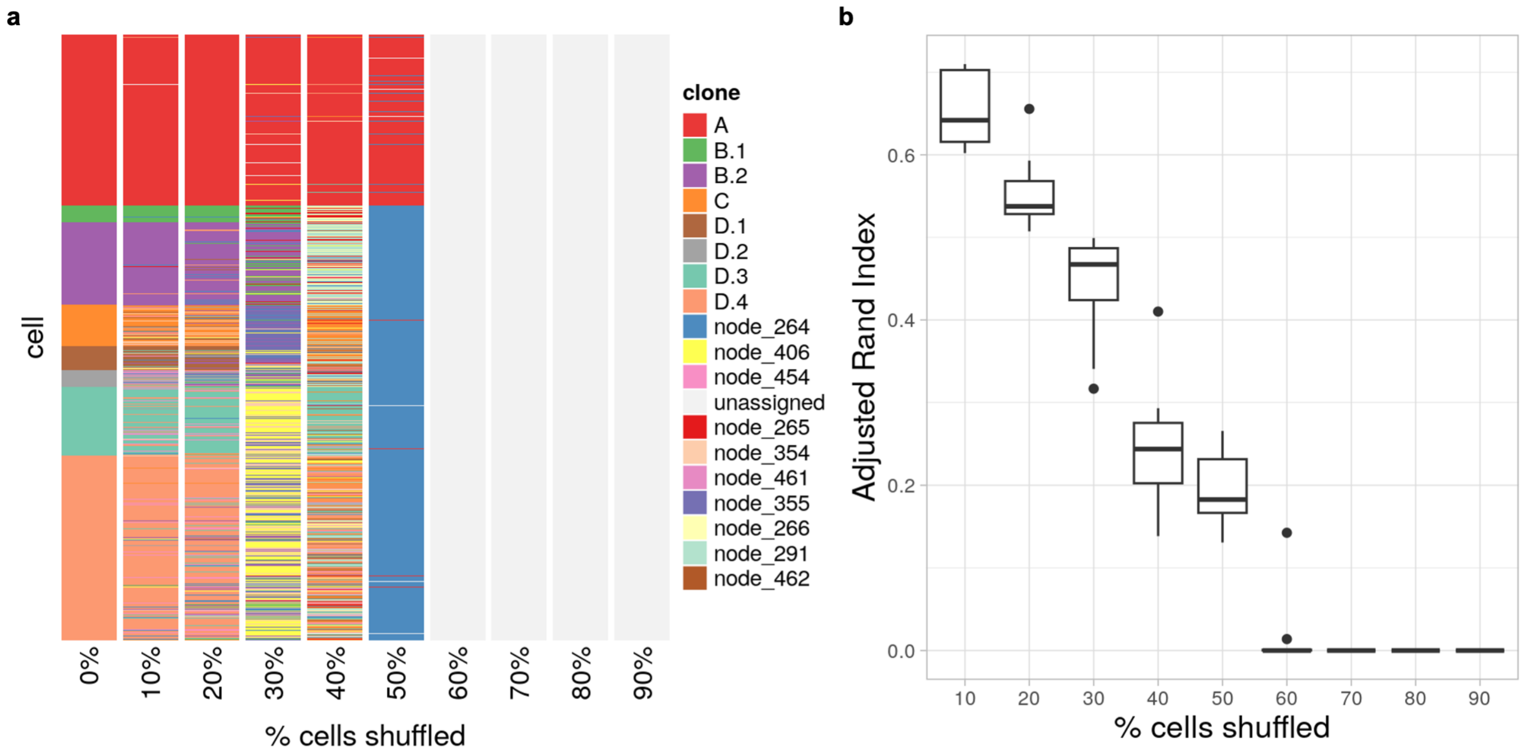

Fig. S9. Clone assignment accuracy of TreeAlign with shuffled phylogenies. a, Heat map of clone assignment in patient 022. Columns represent input phylogenies with certain % of cell labels being randomly shuffled. **b**, Adjusted rand index of clone assignment using shuffled phylogenies in patient 022. Clone assignment results with the original phylogeny were used as ground truth for comparison. Box limits extend from the 25th to 75th percentile, while the middle line represents the median. Whiskers extend to the largest value no further than 1.5 times the inter-quartile range (IQR) from each box hinge. Points beyond the whiskers are outliers.

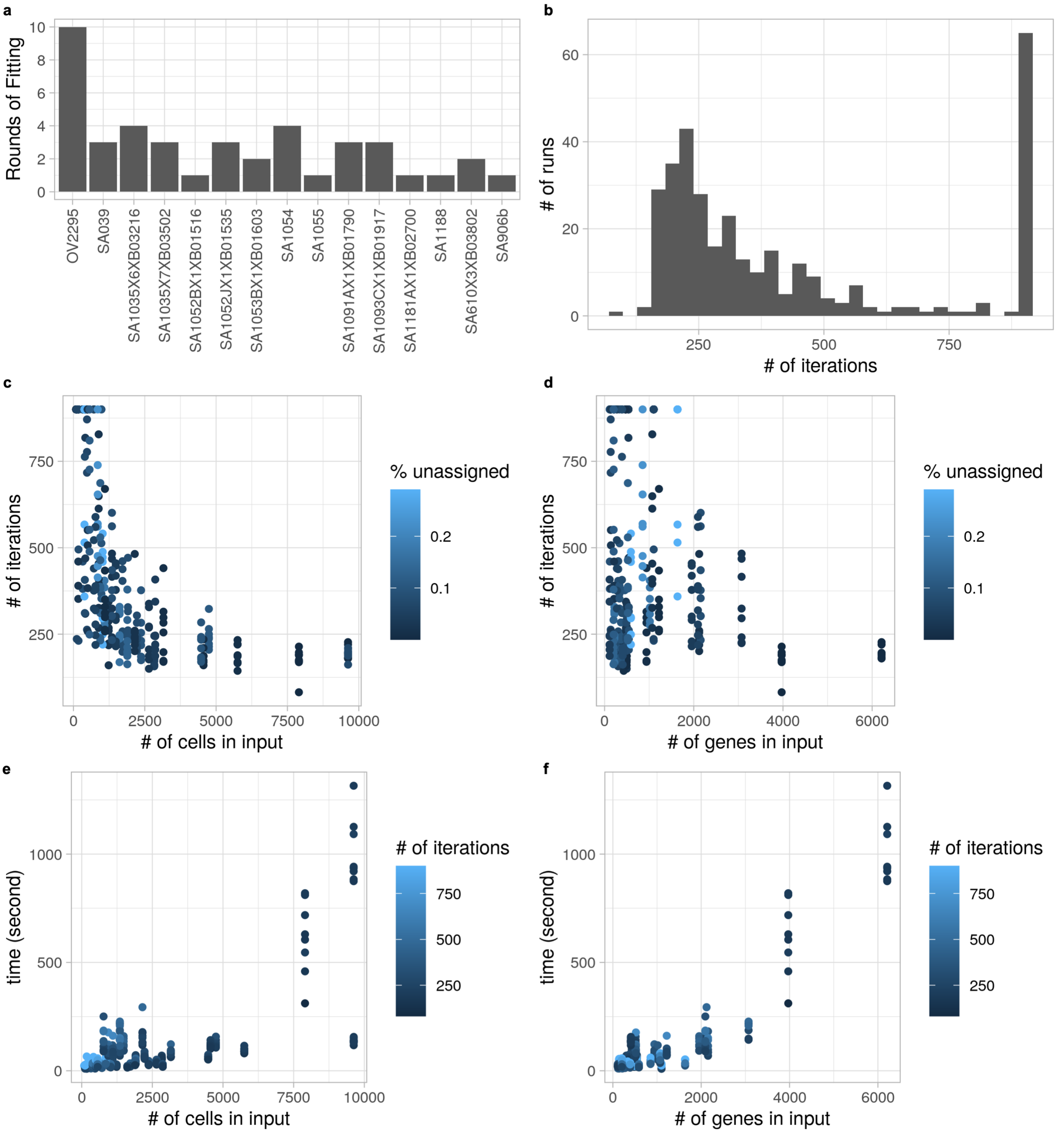

Fig. S10. Inference of integrated TreeAlign in PDXs and cell lines. **a,** rounds of fitting the integrated model with phylogeny input. **b,** Distribution of iterations for each inference run at convergence or before the maximum iteration of 900. **c,** Scatter plot showing the number of iterations and the number of cells in scRNA input for each run colored by frequencies of unassigned cells. **d,** Scatter plot showing the number of iterations and the number of genes in scRNA input for each run. **e,** Scatter plot showing the time to finish for each run as a function of the number of cells in scRNA input. **f,** Scatter plot showing the time to finish for each run as a function of the number of genes in scRNA input.

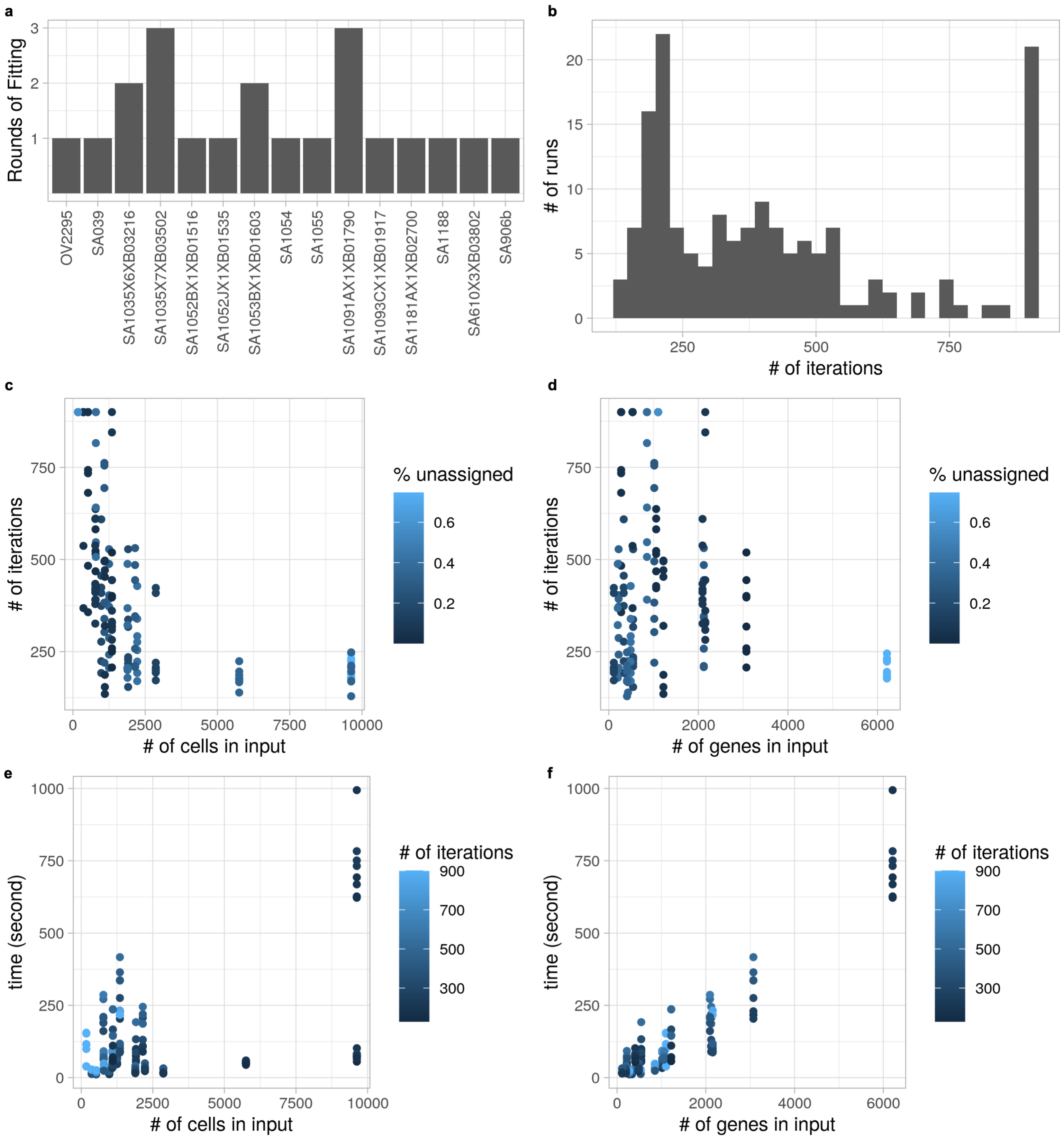

Fig. S11. Inference of total CN TreeAlign in PDXs and cell lines. a, times of fitting the total CN model with phylogeny input. **b,** Distribution of iterations for each inference run at convergence or before the maximum iteration of 900. **c,** Scatter plot showing the number of iterations and the number of cells in scRNA input for each run colored by frequencies of unassigned cells. **d,** Scatter plot showing the number of iterations and the number of genes in scRNA input for each run. **e,** Scatter plot showing the time to finish for each run as a function of the number of cells in scRNA input. **f,** Scatter plot showing the time to finish for each run as a function of the number of genes in scRNA input.

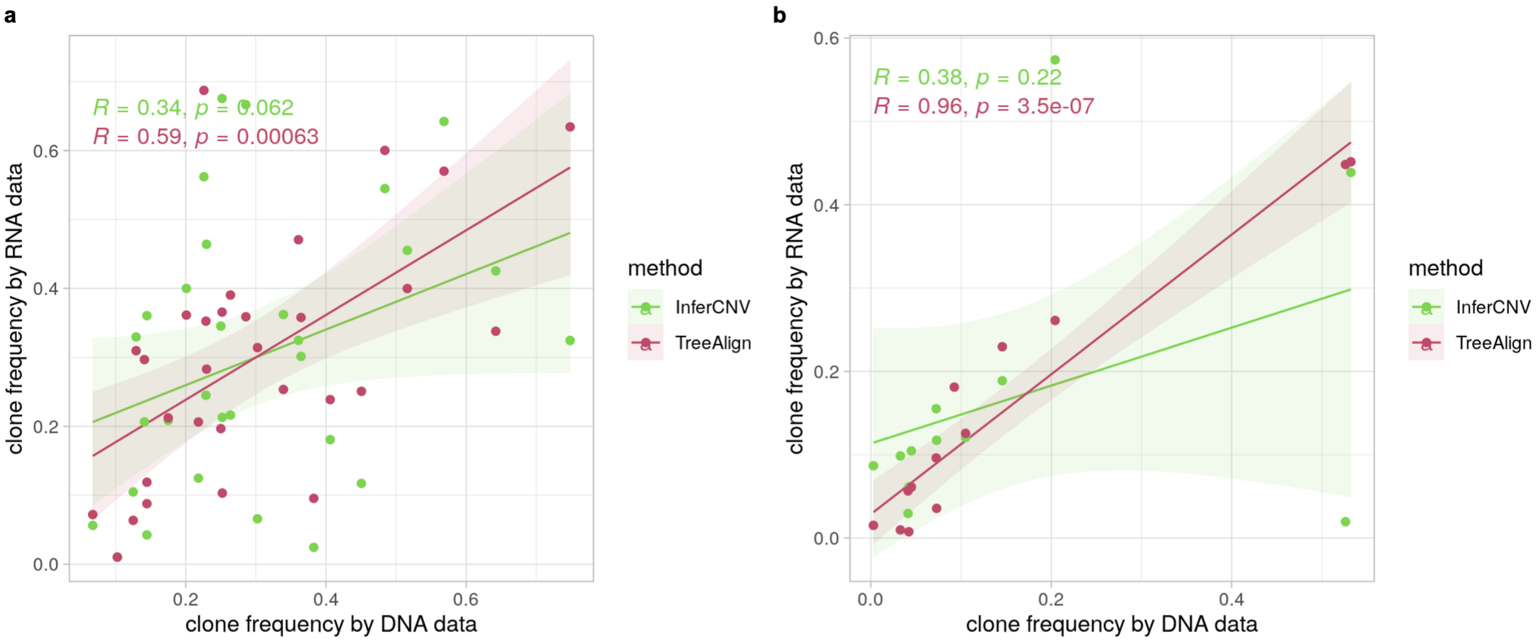

Fig. S12. Compare InferCNV and TreeAlign subclone frequencies. a-b, Correlation between clone frequencies estimated by scRNA-data (x axis) and scDNA-data (y axis) by TreeAlign and InferCNV in **(a)** HSGC PDXs and cell lines and **(b)** patient 022. Pearson correlation coefficients (R) and P values for the linear fit (Two-sided Student's t-test) are shown.

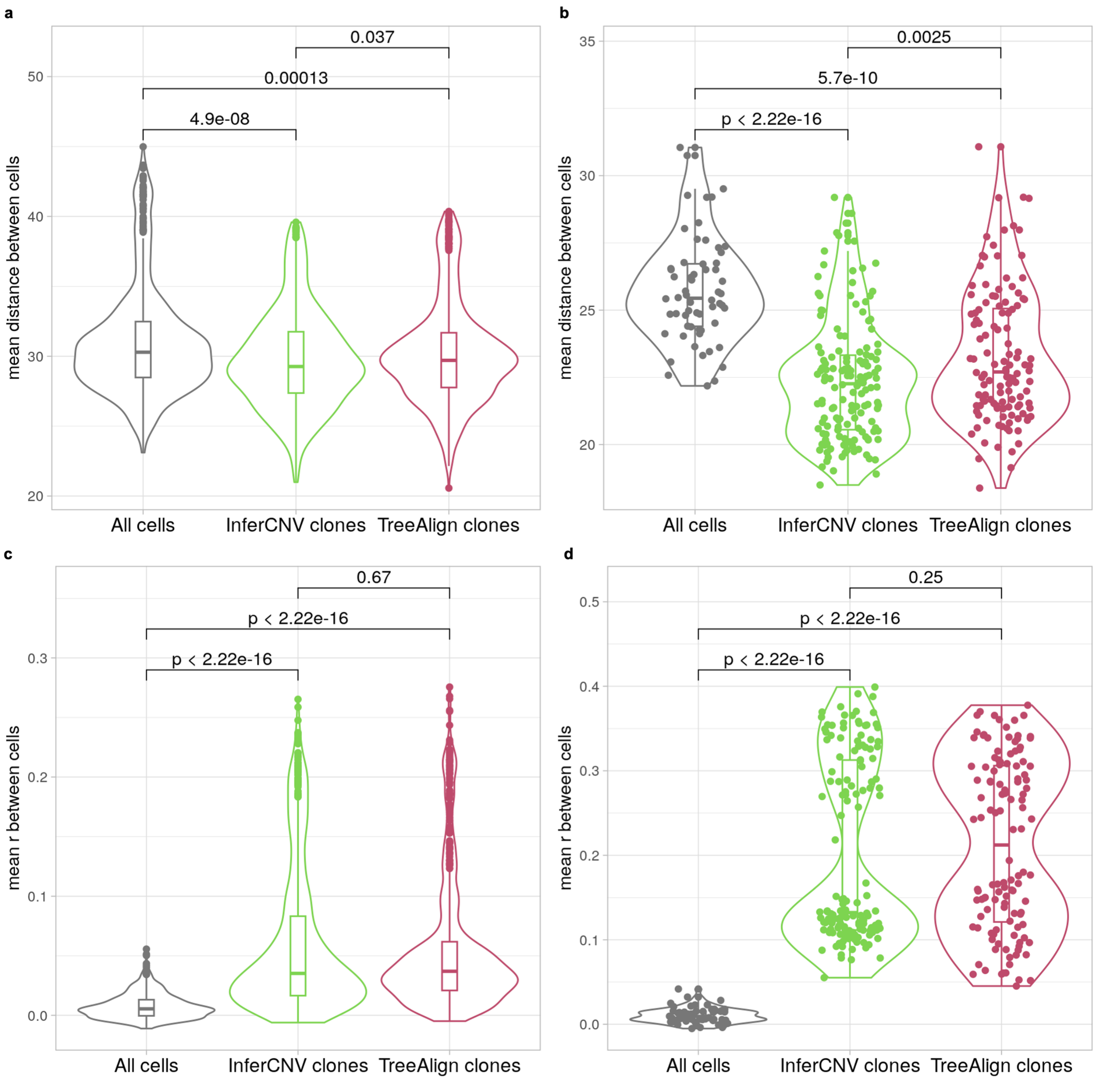

Fig. S13. Subclonal transcriptional diversity. a-b, mean Euclidean distance between cells in scRNA-data sampled across or within subclones for **(a)** HSGC PDXs and cell lines and **(b)** patient 022. **c-d,** mean Pearson correlation coefficient between cells in scRNA-data sampled across or within subclones for **(c)** HSGC PDXs and cell lines and **(d)** patient 022. (Two-sided Wilcoxon signed-rank test). For the box plots, box limits extend from the 25th to 75th percentile, while the middle line represents the median. Whiskers extend to the largest value no further than 1.5 times the inter-quartile range (IQR) from each box hinge. Points beyond the whiskers are outliers.

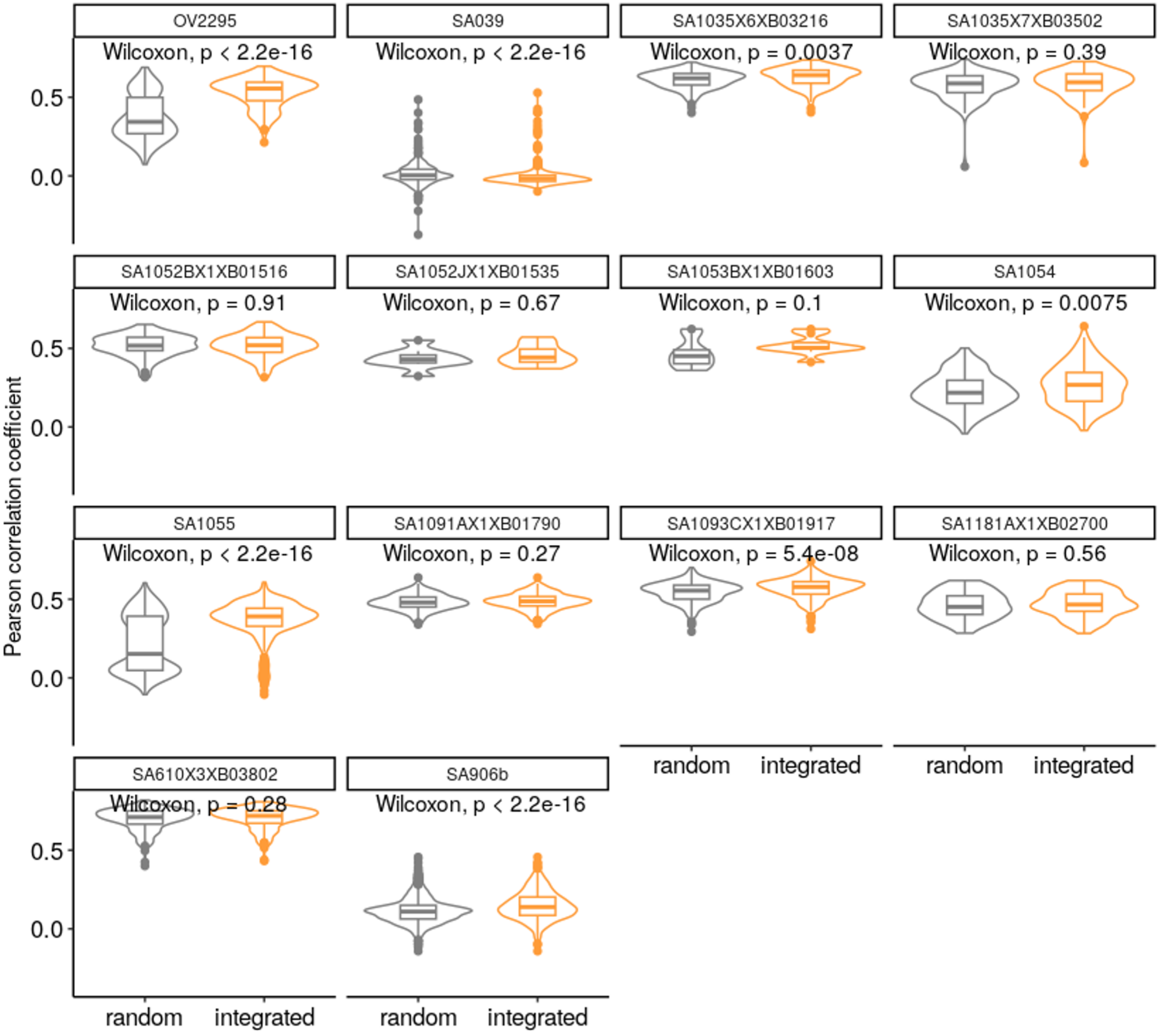

Fig. S14. Integrated TreeAlign has improved clone assignment performance compared to total CN TreeAlign. Distribution of Pearson correlation coefficients (R) between scDNA estimated total copy number and InferCNV corrected expression for unassigned cells from total CN model. Left, correlation distribution calculated by comparing InferCNV profiles to CN profiles of a random subclone; Right, correlation distribution calculated by comparing InferCNV profiles to CN profiles of subclones assigned by integrated TreeAlign. Each panel represents results from a tumor sample/cell line. (Two-sided Wilcoxon signed-rank test). For the box plots, box limits extend from the 25th to 75th percentile, while the middle line represents the median. Whiskers extend to the largest value no further than 1.5 times the inter-quartile range (IQR) from each box hinge. Points beyond the whiskers are outliers.

a

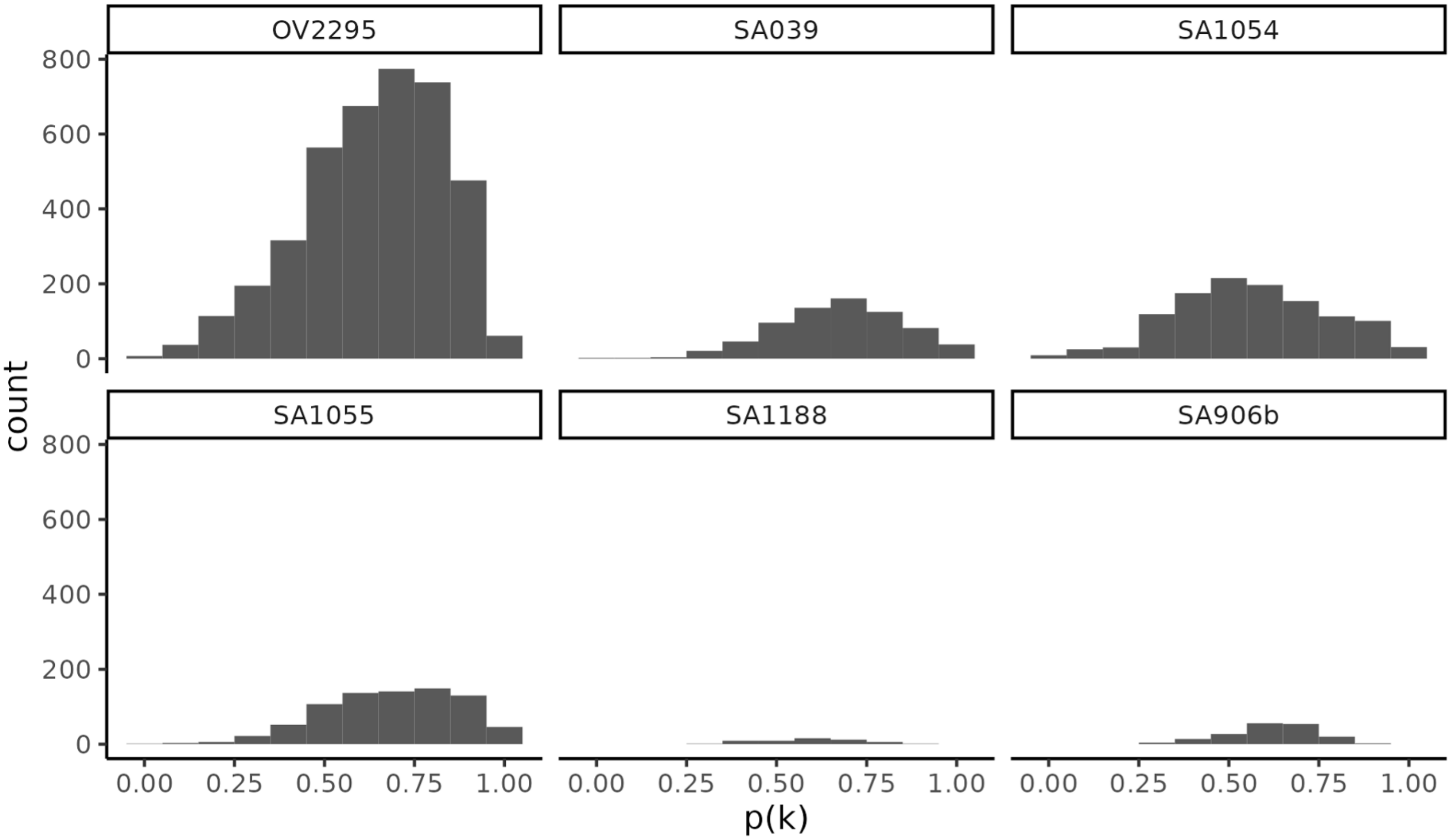

b

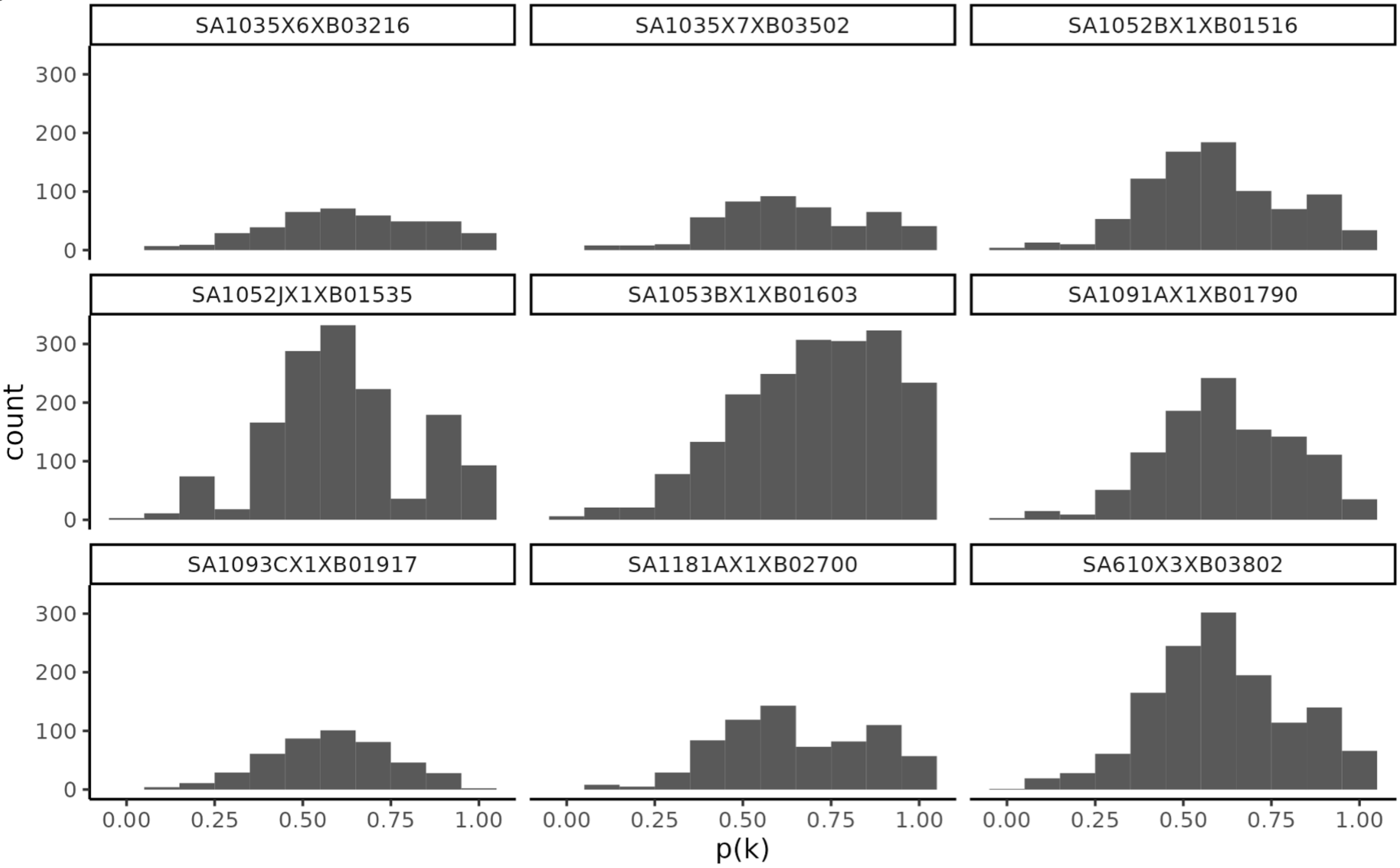

Fig. S15. Distribution of $p(k)$ in HGSC PDXs and cell lines. a, Distribution of $p(k)$ in hTERT-184 and control cell lines. **b,** Distribution of $p(k)$ in PDXs.

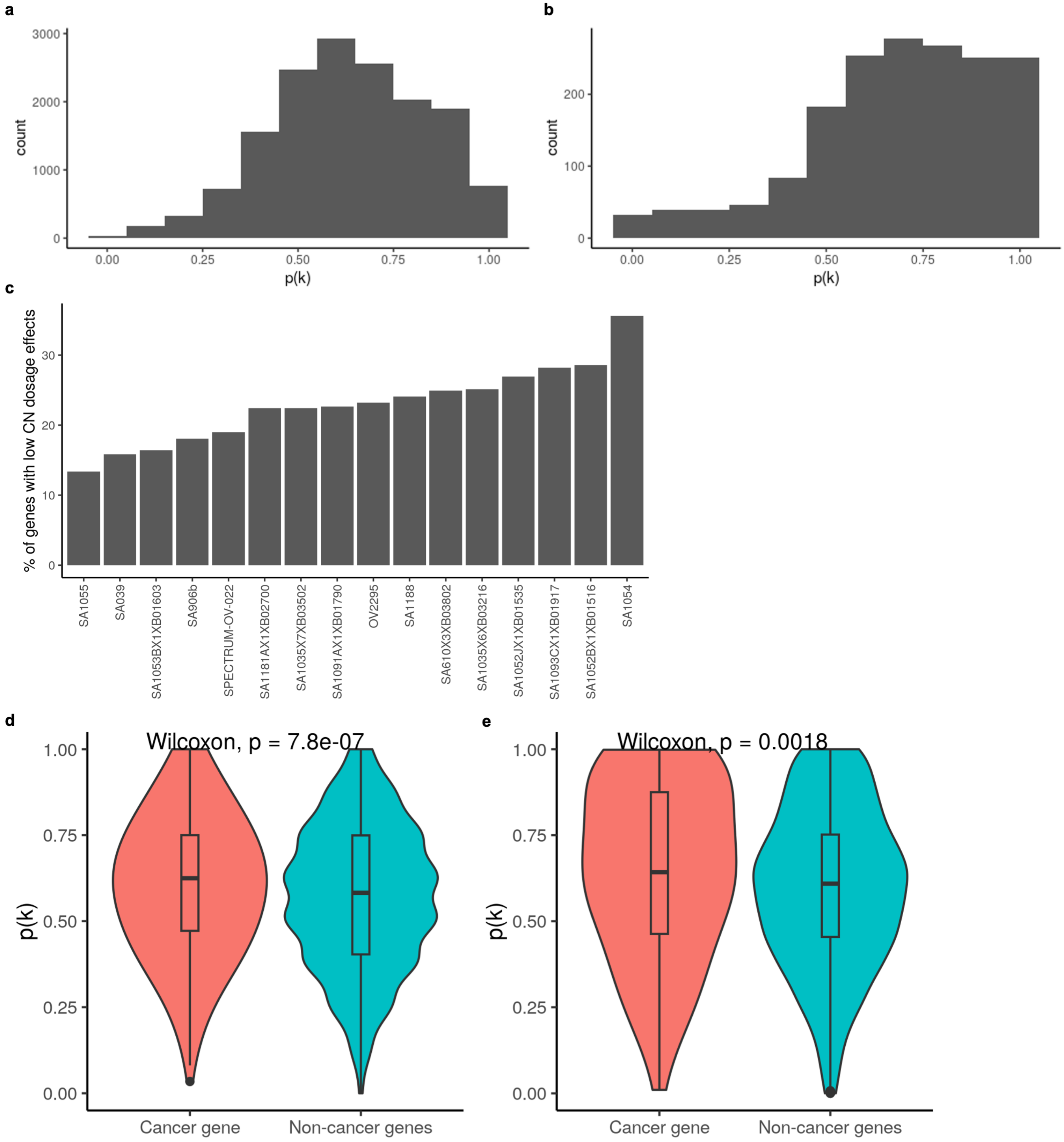

Fig. S16. Low $p(k)$ genes in patient 022, HGSC PDXs and cell lines. a, Distribution of $p(k)$ in PDXs and cell lines. **b,** Distribution of $p(k)$ in patient 022. **c,** Proportions of genes with low CN dosage effects ($p(k) < 0.5$) in PDXs and cell lines. **d-e,** $p(k)$ for cancer genes and non-cancer genes in **(d)** PDXs and cell lines and **(e)** patient 022. (Two-sided Wilcoxon signed-rank test). For the box plots, box limits extend from the 25th to 75th percentile, while the middle line represents the median. Whiskers extend to the largest value no further than 1.5 times the inter-quartile range (IQR) from each box hinge. Points beyond the whiskers are outliers.

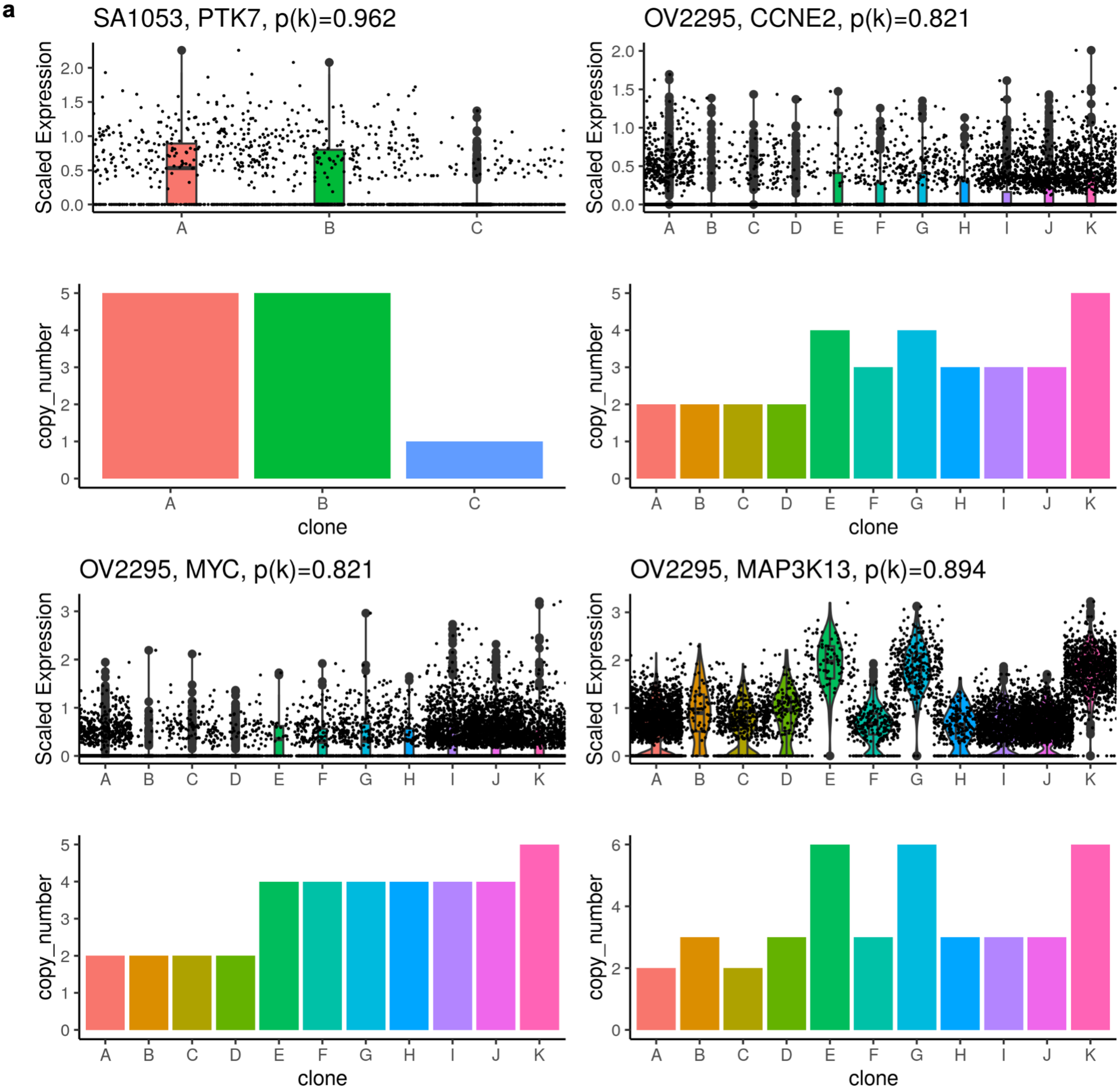

Fig. S17. Examples of high $p(k)$ gene. a, Examples of genes with high level amplifications and high CN dosage effects.

a

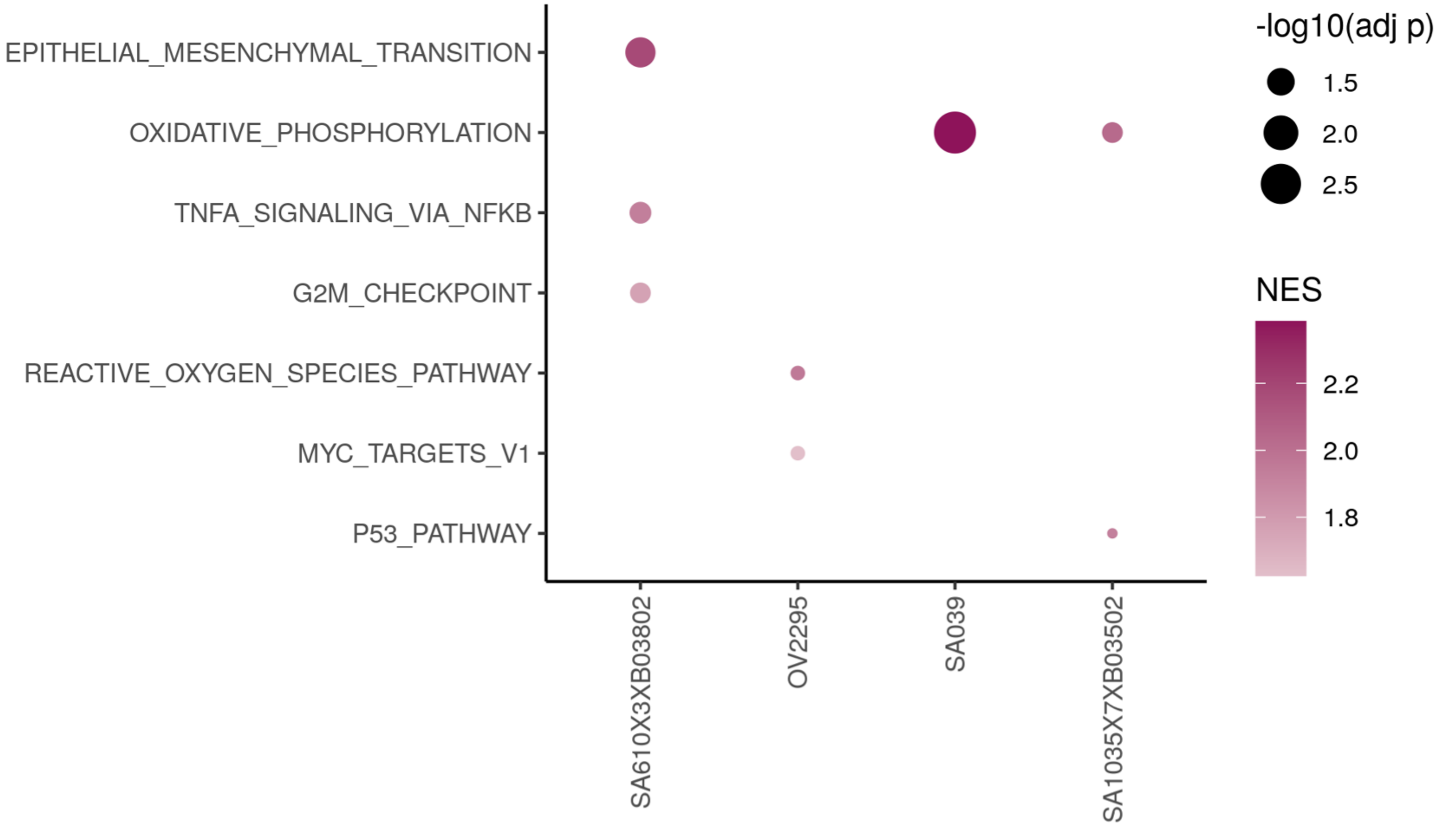

b

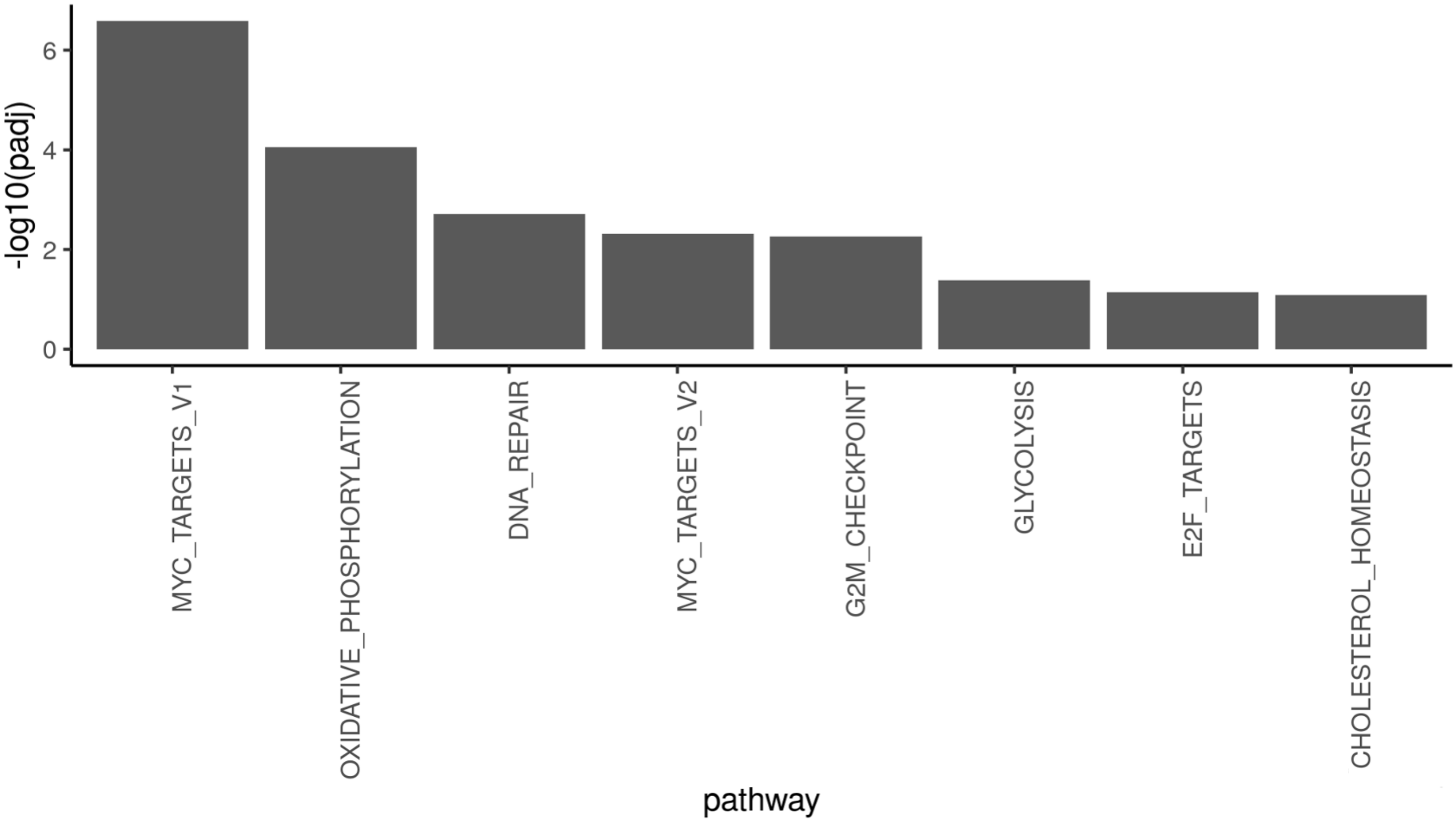

Fig. S18. Gene set enrichment analysis of low p(k) genes. a, Dot plot showing significantly enriched pathways in low p(k) genes. **b,** Significantly enriched pathways in low $p(k)$ genes from all PDXs and cell lines. $p(k)$ from all samples were combined before performing gene set enrichment analysis.

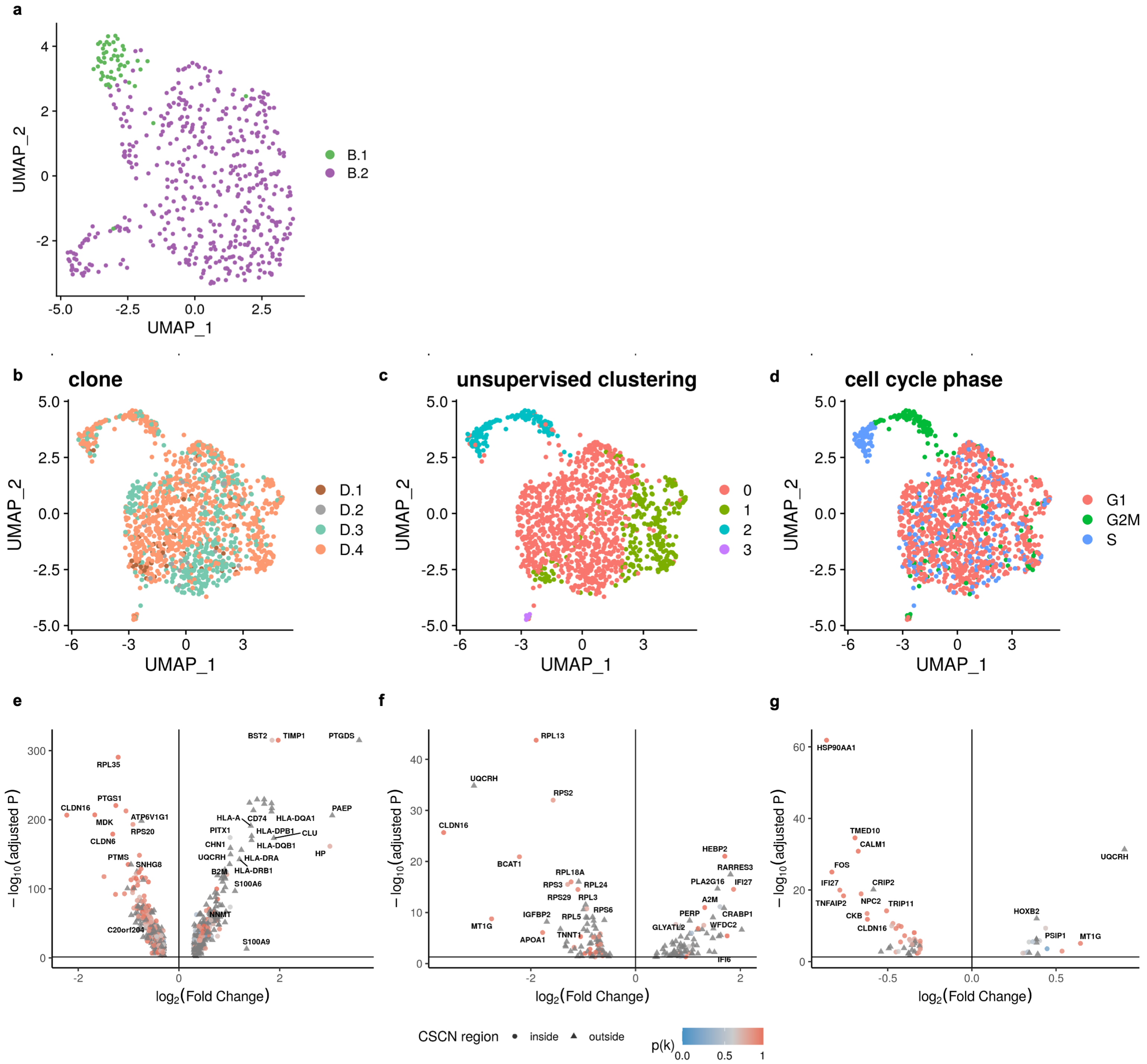

Fig. S19. Differentially expressed genes between subclones in patient 022. a, UMAP plot of expression profiles of clone B.1 and B.2 in patient 022. **b,** UMAP plot of expression profiles of clone D.1, D.2, D.3 and D.4 in patient 022 colored by clone assignments. **c,** UMAP plot of expression profiles of clone D in patient 022 colored by Louvain unsupervised clustering. **d,** UMAP plot of expression profiles of clone D in patient 022 colored by cell cycle phase. **e,** Differentially expressed genes between clone A and clone B-D. **f,** Differentially expressed genes between cells in clone B.1 and B.2. **g,** Differentially expressed genes between cells in clone D.4 and D.1 - D.3.

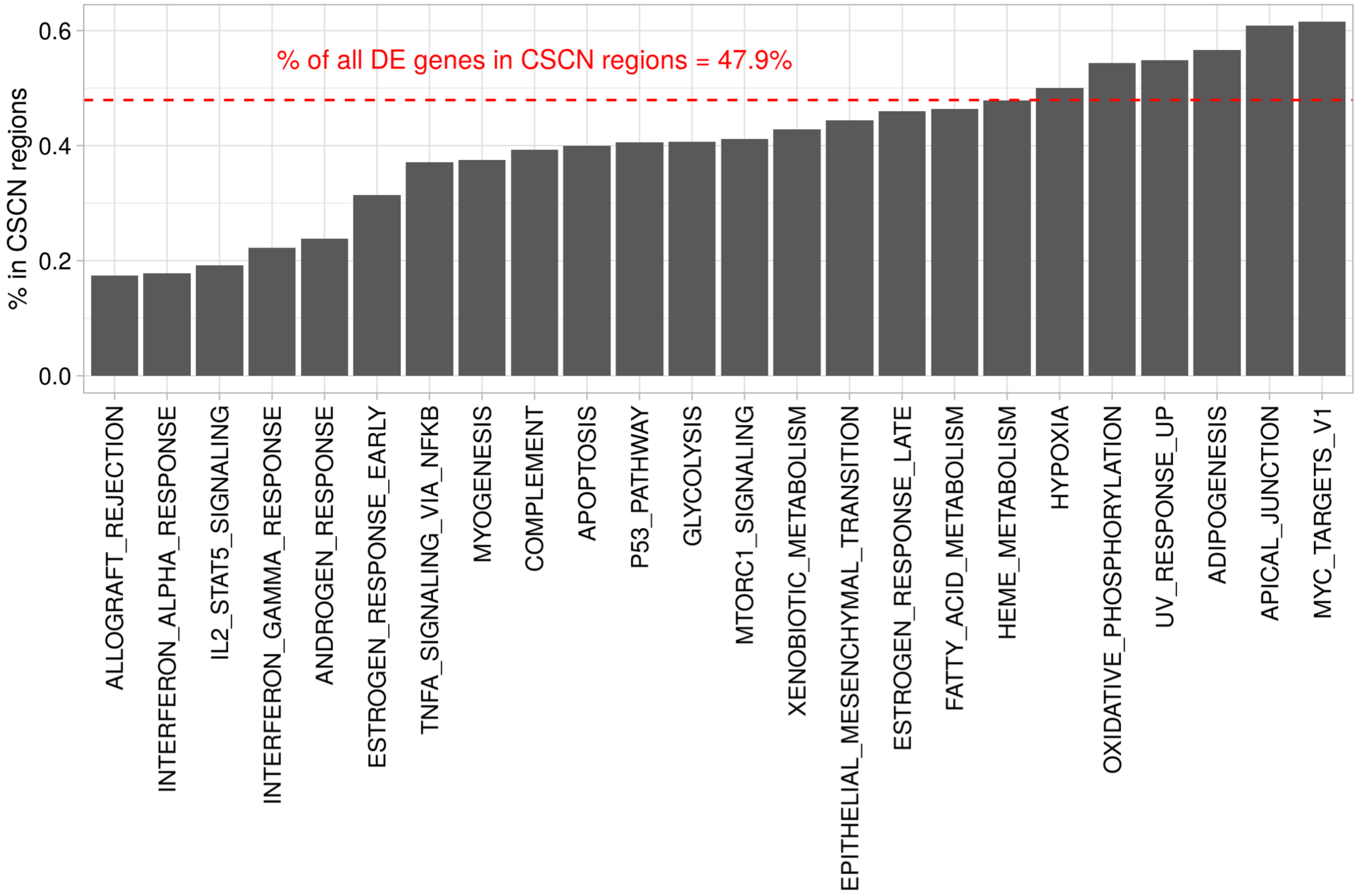

Fig. S20. Frequencies of DE genes in CSCN regions summarized by Hallmark pathways.

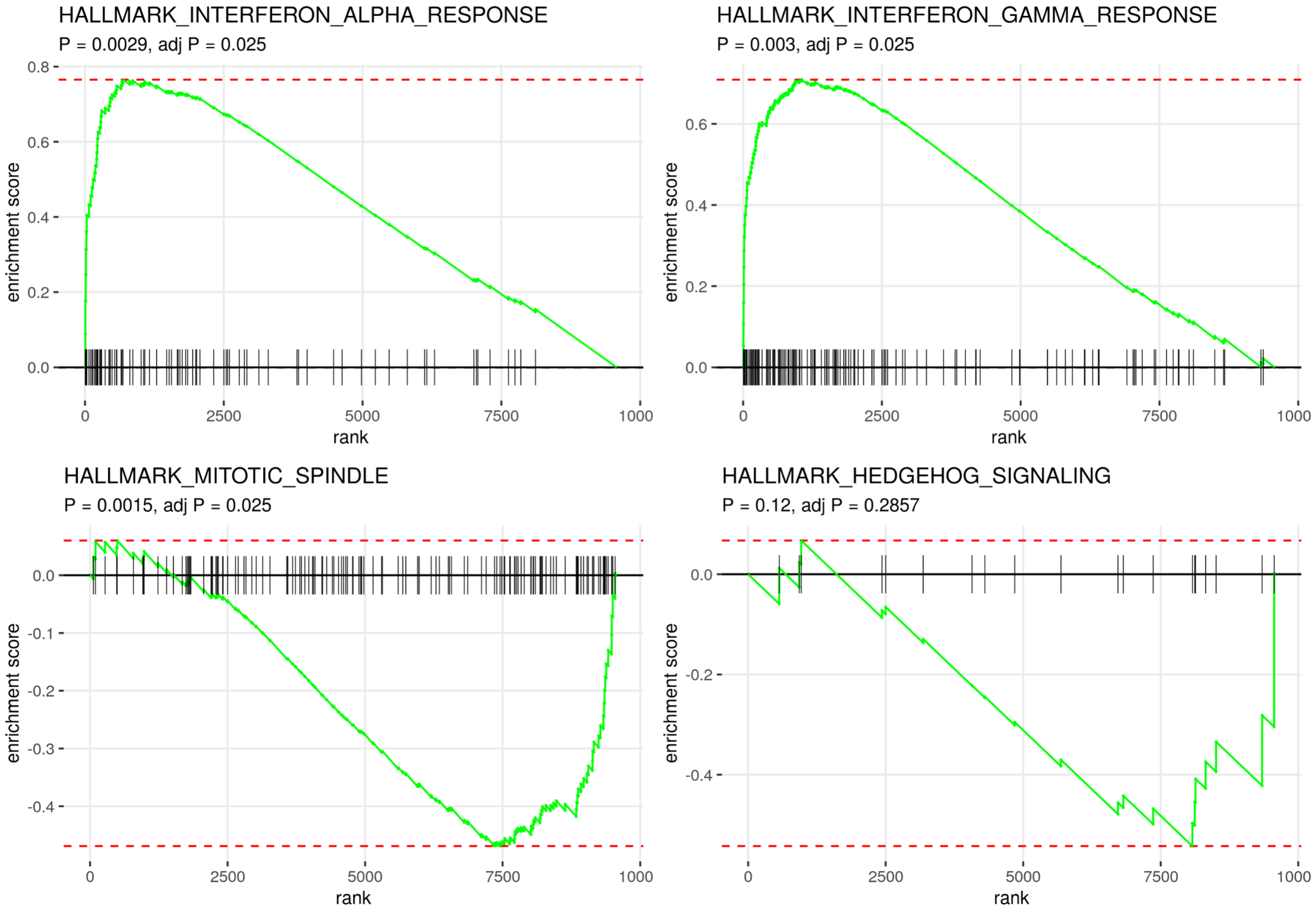

Fig. S21. Enriched and depleted pathways in clone A compared to other clones in patient 022.

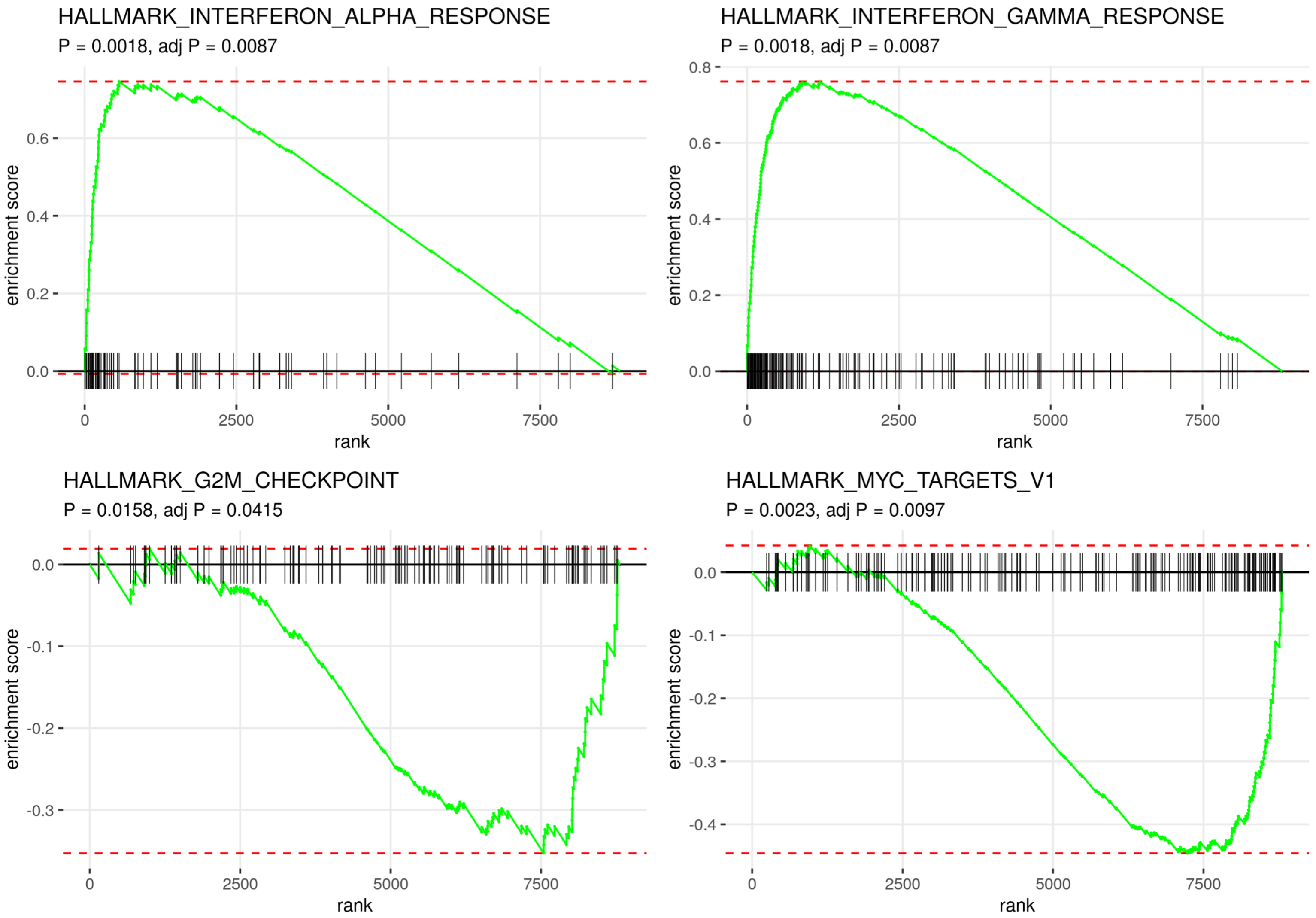

Fig. S22. Enriched and depleted pathways in clone B.1 compared to clone B.2 in patient 022.

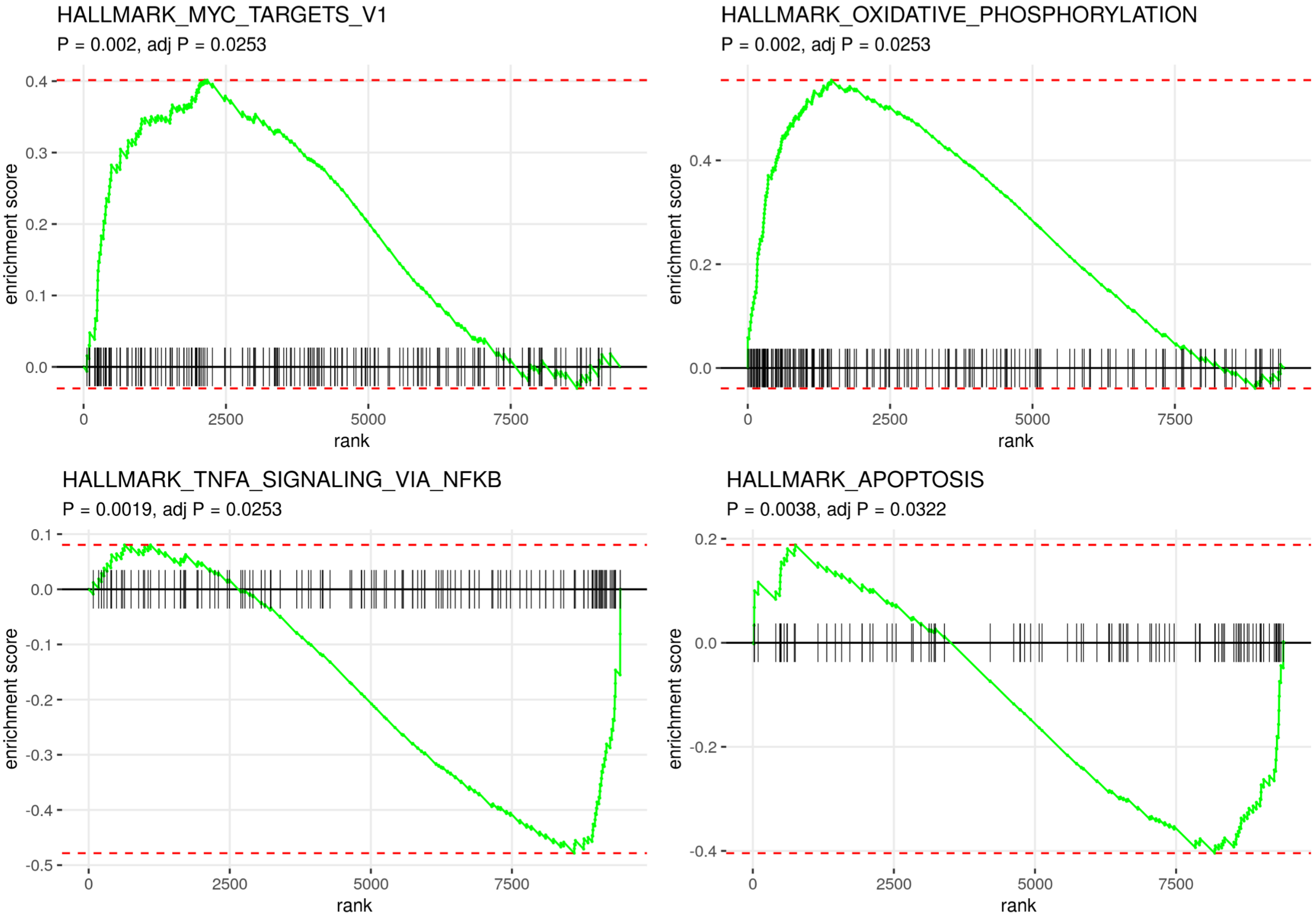

Fig. S23. Enriched and depleted pathways in clone D.4 compared to the rest of cells in clone D in patient 022.

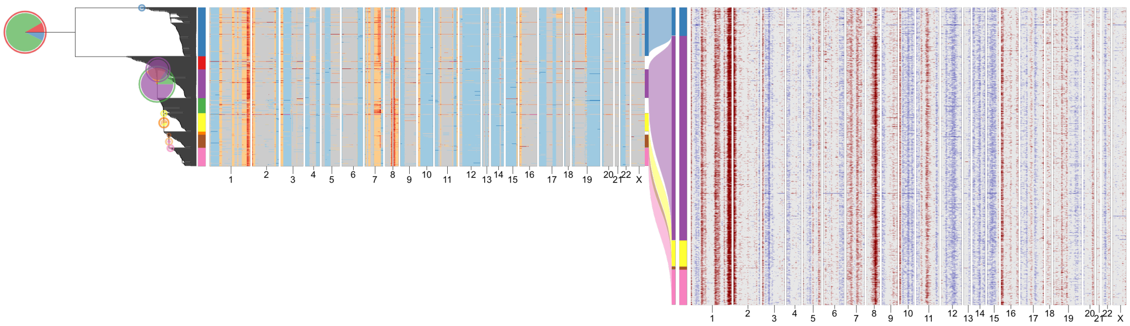

Fig. S24. Integrated model of TreeAlign assigns expression profiles of SA1035X6XB03216 to phylogeny. Heat maps of copy number profiles from scDNA (left) and InferCNV corrected expression profiles from scRNA (right). The Sankey chart in the middle shows clone assignment from expression profiles to copy number based clones by integrated TreeAlign.

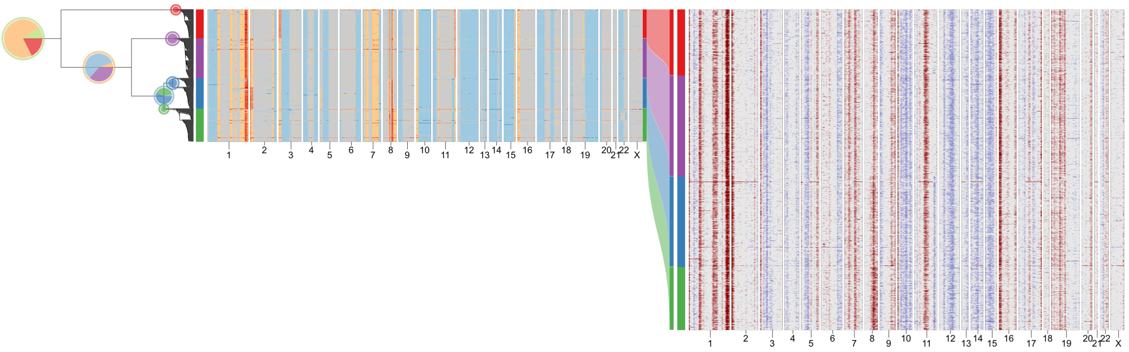

Fig. S25. Integrated model of TreeAlign assigns expression profiles of SA1035X7XB03502 to phylogeny. Heat maps of copy number profiles from scDNA (left) and InferCNV corrected expression profiles from scRNA (right). The Sankey chart in the middle shows clone assignment from expression profiles to copy number based clones by integrated TreeAlign.

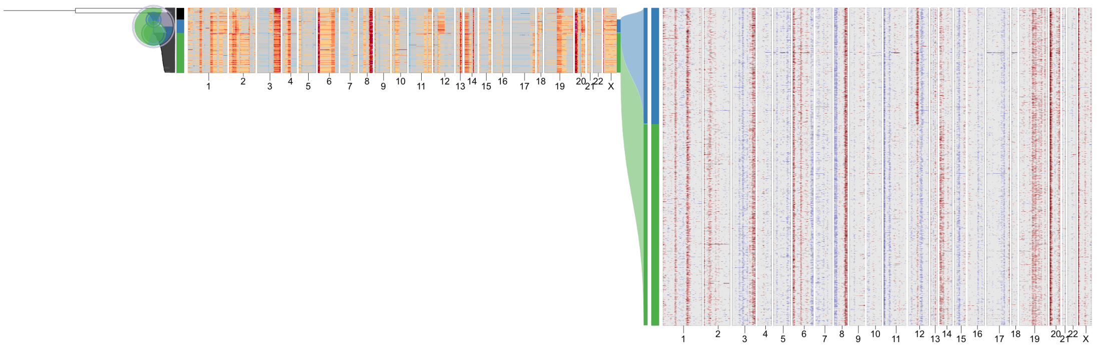

Fig. S26. Integrated model of TreeAlign assigns expression profiles of SA1052BX1XB01516 to phylogeny. Heat maps of copy number profiles from scDNA (left) and InferCNV corrected expression profiles from scRNA (right). The Sankey chart in the middle shows clone assignment from expression profiles to copy number based clones by integrated TreeAlign.

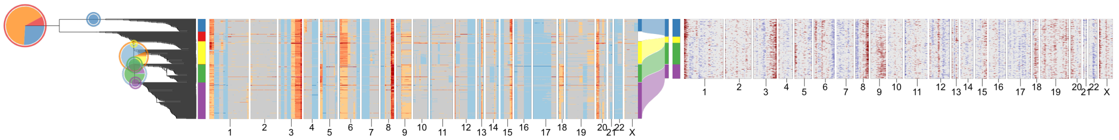

Fig. S27. Integrated model of TreeAlign assigns expression profiles of SA1052JX1XB01535 to phylogeny. Heat maps of copy number profiles from scDNA (left) and InferCNV corrected expression profiles from scRNA (right). The Sankey chart in the middle shows clone assignment from expression profiles to copy number based clones by integrated TreeAlign.

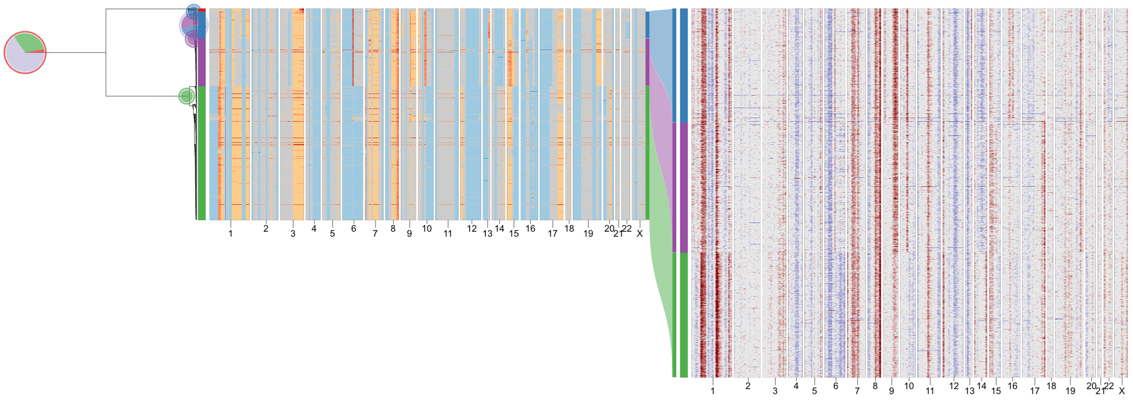

Fig. S28. Integrated model of TreeAlign assigns expression profiles of SA1053BX1XB01603 to phylogeny. Heat maps of copy number profiles from scDNA (left) and InferCNV corrected expression profiles from scRNA (right). The Sankey chart in the middle shows clone assignment from expression profiles to copy number based clones by integrated TreeAlign.

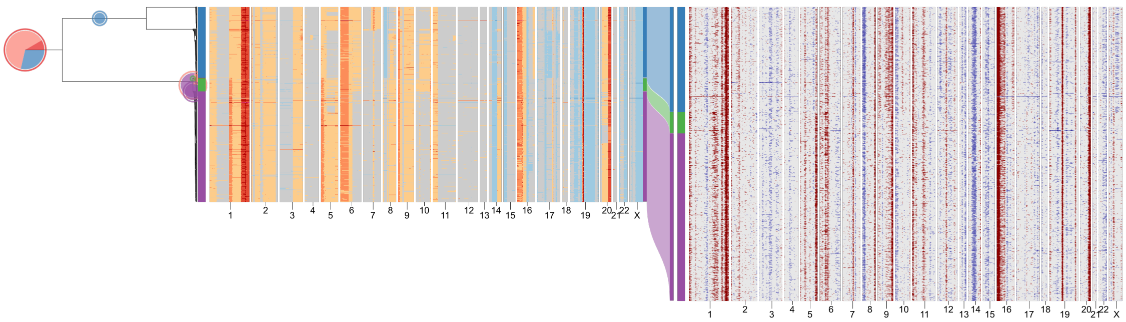

Fig. S29. Integrated model of TreeAlign assigns expression profiles of SA1091AX1XB01790 to phylogeny. Heat maps of copy number profiles from scDNA (left) and InferCNV corrected expression profiles from scRNA (right). The Sankey chart in the middle shows clone assignment from expression profiles to copy number based clones by integrated TreeAlign.

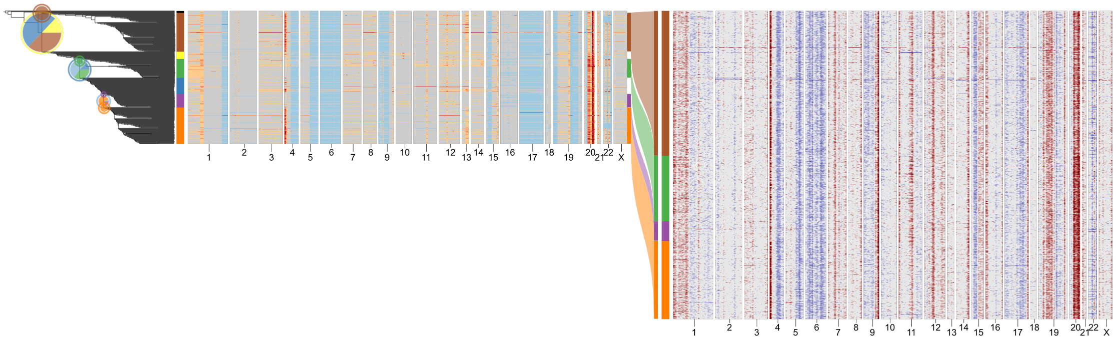

Fig. S30. Integrated model of TreeAlign assigns expression profiles of SA1093CX1XB01917 to phylogeny. Heat maps of copy number profiles from scDNA (left) and InferCNV corrected expression profiles from scRNA (right). The Sankey chart in the middle shows clone assignment from expression profiles to copy number based clones by integrated TreeAlign.

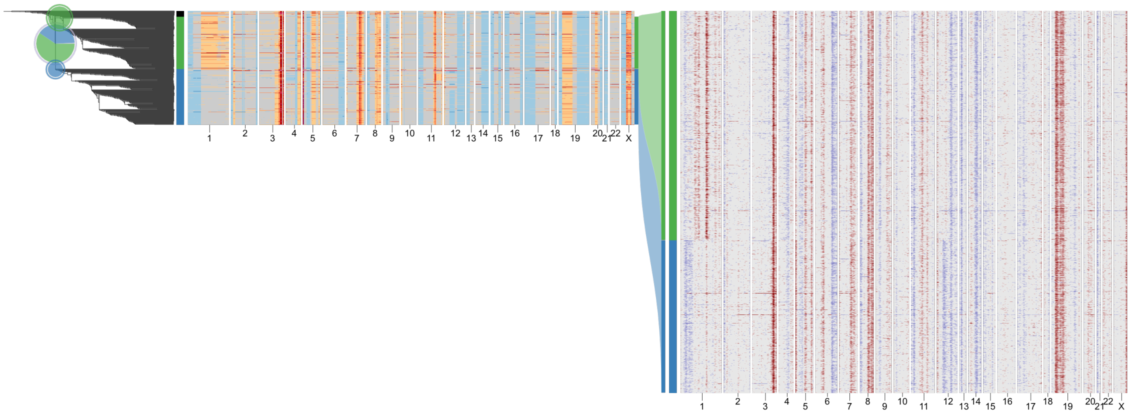

Fig. S31. Integrated model of TreeAlign assigns expression profiles of SA1181AX1XB02700 to phylogeny. Heat maps of copy number profiles from scDNA (left) and InferCNV corrected expression profiles from scRNA (right). The Sankey chart in the middle shows clone assignment from expression profiles to copy number based clones by integrated TreeAlign.

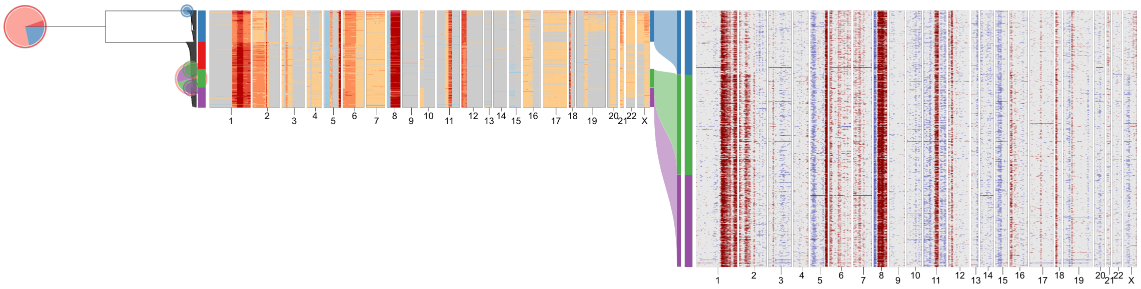

Fig. S32. Integrated model of TreeAlign assigns expression profiles of SA610X3XB03802 to phylogeny. Heat maps of copy number profiles from scDNA (left) and InferCNV corrected expression profiles from scRNA (right). The Sankey chart in the middle shows clone assignment from expression profiles to copy number based clones by integrated TreeAlign.

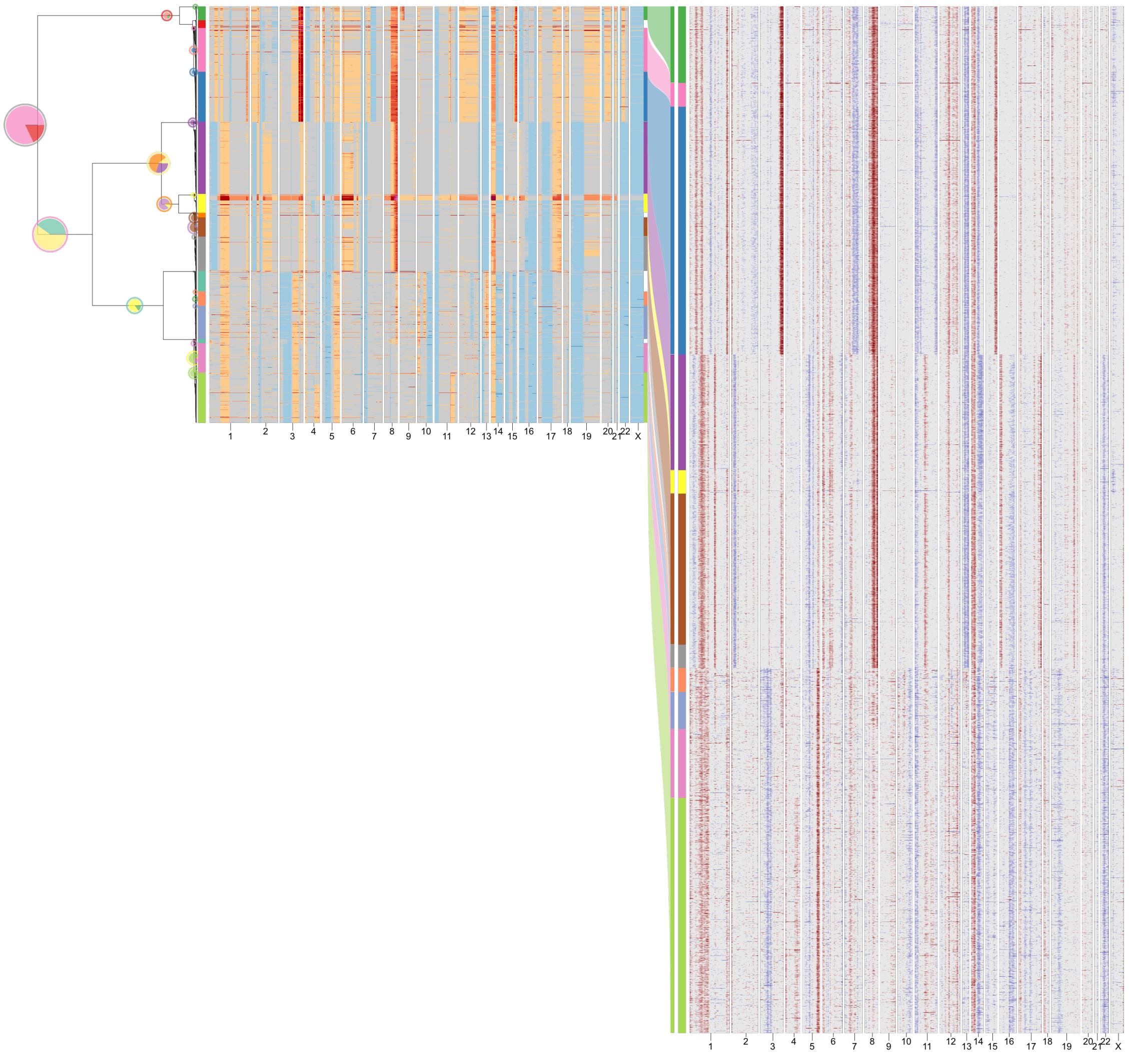

Fig. S33. Integrated model of TreeAlign assigns expression profiles of OV2295 to phylogeny. Heat maps of copy number profiles from scDNA (left) and InferCNV corrected expression profiles from scRNA (right). The Sankey chart in the middle shows clone assignment from expression profiles to copy number based clones by integrated TreeAlign.

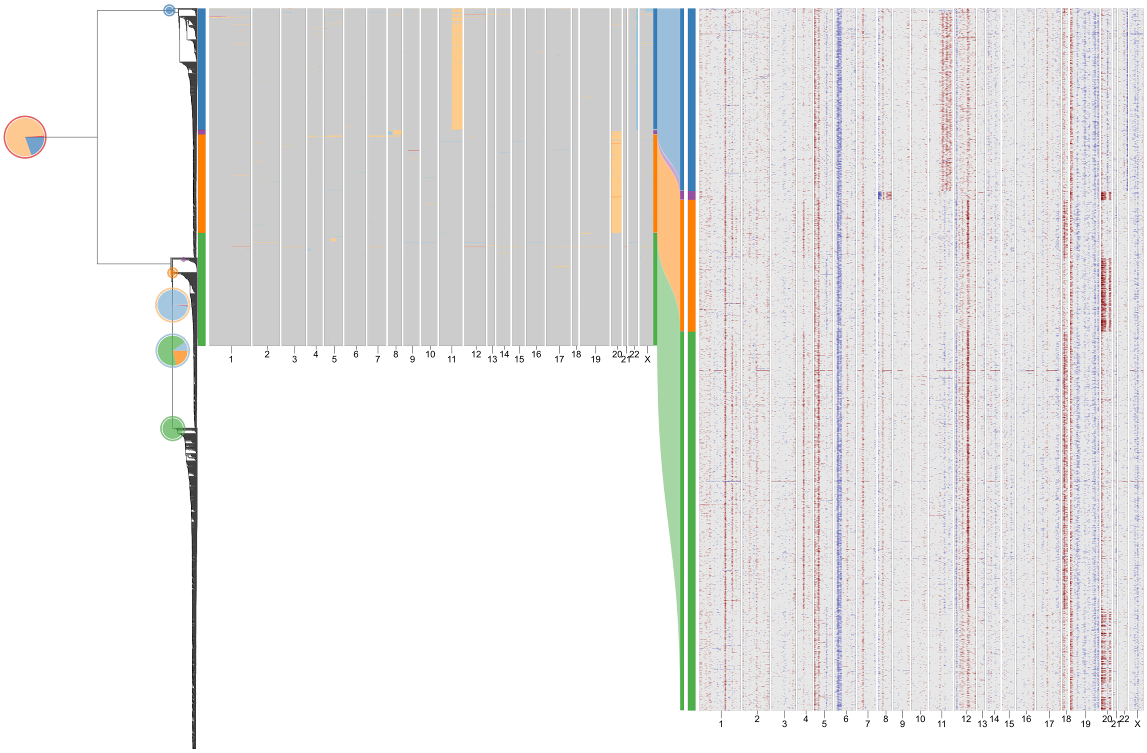

Fig. S34. Integrated model of TreeAlign assigns expression profiles of SA039 to phylogeny. Heat maps of copy number profiles from scDNA (left) and InferCNV corrected expression profiles from scRNA (right). The Sankey chart in the middle shows clone assignment from expression profiles to copy number based clones by integrated TreeAlign.

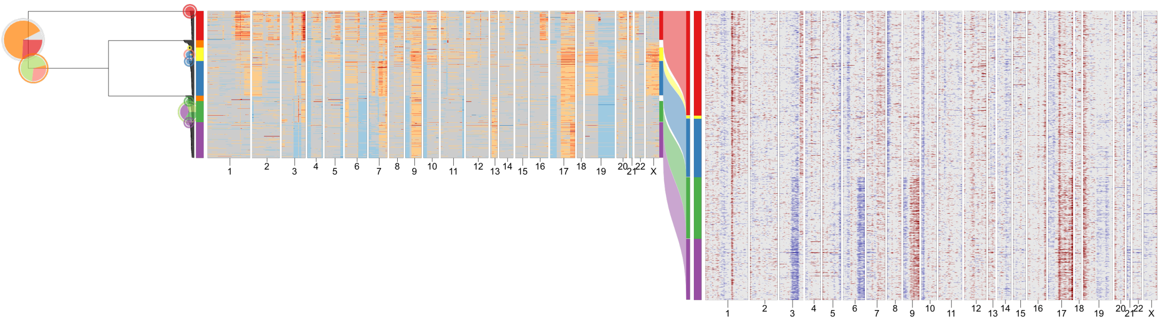

Fig. S35. Integrated model of TreeAlign assigns expression profiles of SA1054 to phylogeny. Heat maps of copy number profiles from scDNA (left) and InferCNV corrected expression profiles from scRNA (right). The Sankey chart in the middle shows clone assignment from expression profiles to copy number based clones by integrated TreeAlign.

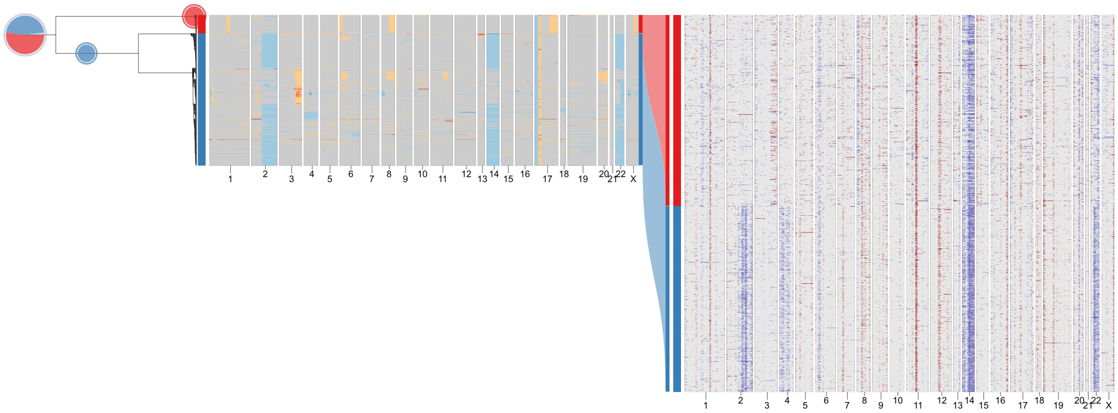

Fig. S36. Integrated model of TreeAlign assigns expression profiles of SA1055 to phylogeny. Heat maps of copy number profiles from scDNA (left) and InferCNV corrected expression profiles from scRNA (right). The Sankey chart in the middle shows clone assignment from expression profiles to copy number based clones by integrated TreeAlign.

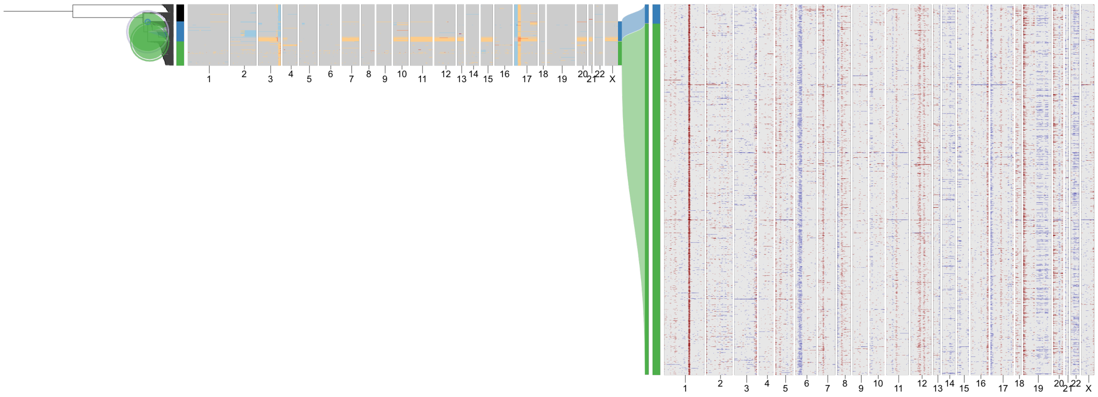

Fig. S37. Integrated model of TreeAlign assigns expression profiles of SA1188 to phylogeny. Heat maps of copy number profiles from scDNA (left) and InferCNV corrected expression profiles from scRNA (right). The Sankey chart in the middle shows clone assignment from expression profiles to copy number based clones by integrated TreeAlign.

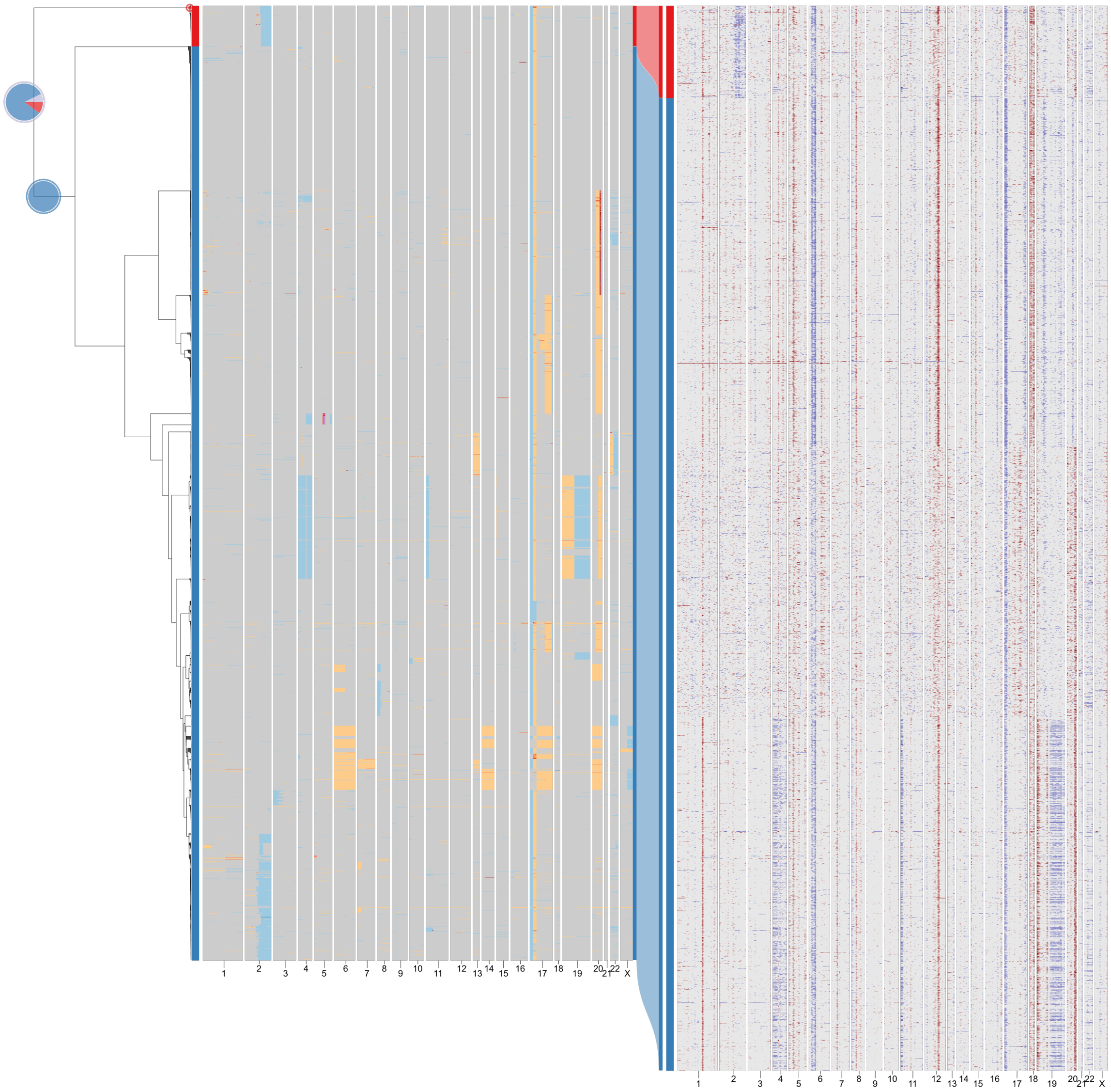

Fig. S38. Integrated model of TreeAlign assigns expression profiles of SA906b to phylogeny. Heat maps of copy number profiles from scDNA (left) and InferCNV corrected expression profiles from scRNA (right). The Sankey chart in the middle shows clone assignment from expression profiles to copy number based clones by integrated TreeAlign.

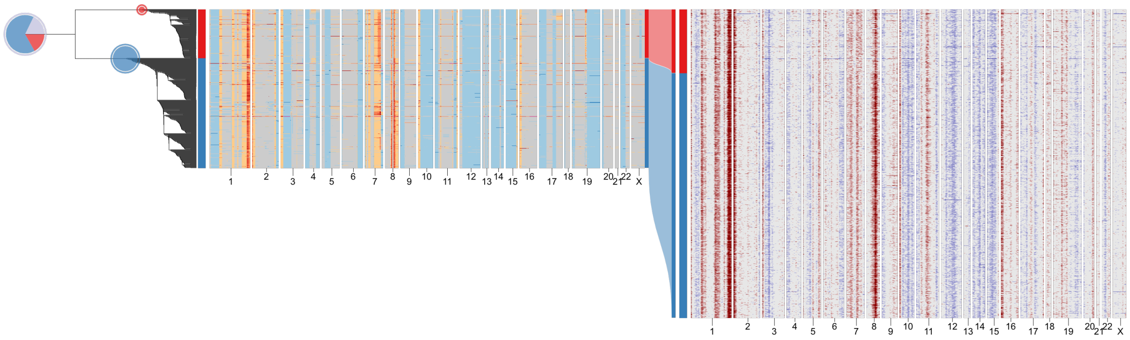

Fig. S39. Total CN model of TreeAlign assigns expression profiles of SA1035X6XB03216 to phylogeny. Heat maps of copy number profiles from scDNA (left) and InferCNV corrected expression profiles from scRNA (right). The Sankey chart in the middle shows clone assignment from expression profiles to copy number based clones by total CN TreeAlign.

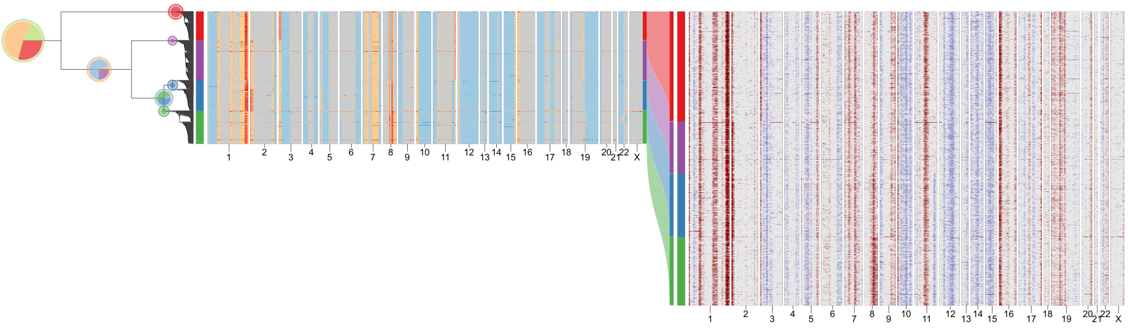

Fig. S40. Total CN model of TreeAlign assigns expression profiles of SA1035X7XB03502 to phylogeny. Heat maps of copy number profiles from scDNA (left) and InferCNV corrected expression profiles from scRNA (right). The Sankey chart in the middle shows clone assignment from expression profiles to copy number based clones by total CN TreeAlign.

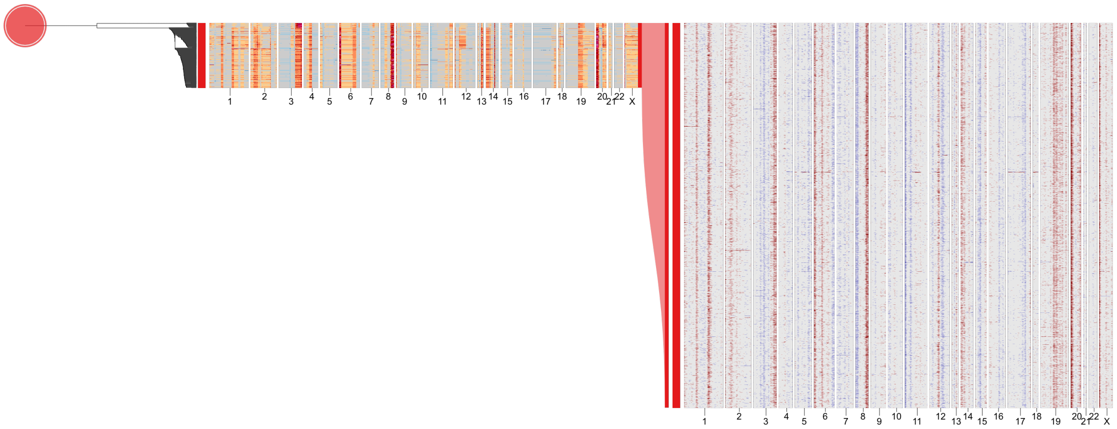

Fig. S41. Total CN model of TreeAlign assigns expression profiles of SA1052BX1XB01516 to phylogeny. Heat maps of copy number profiles from scDNA (left) and InferCNV corrected expression profiles from scRNA (right). The Sankey chart in the middle shows clone assignment from expression profiles to copy number based clones by total CN TreeAlign.

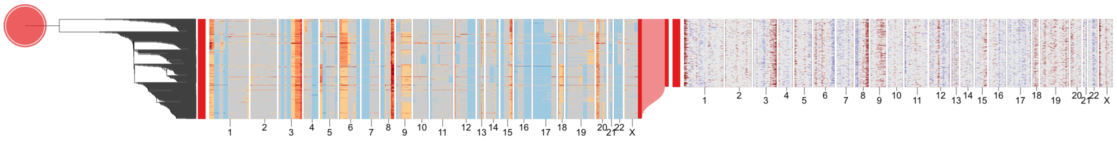

Fig. S42. Total CN model of TreeAlign assigns expression profiles of SA1052JX1XB01535 to phylogeny. Heat maps of copy number profiles from scDNA (left) and InferCNV corrected expression profiles from scRNA (right). The Sankey chart in the middle shows clone assignment from expression profiles to copy number based clones by total CN TreeAlign.

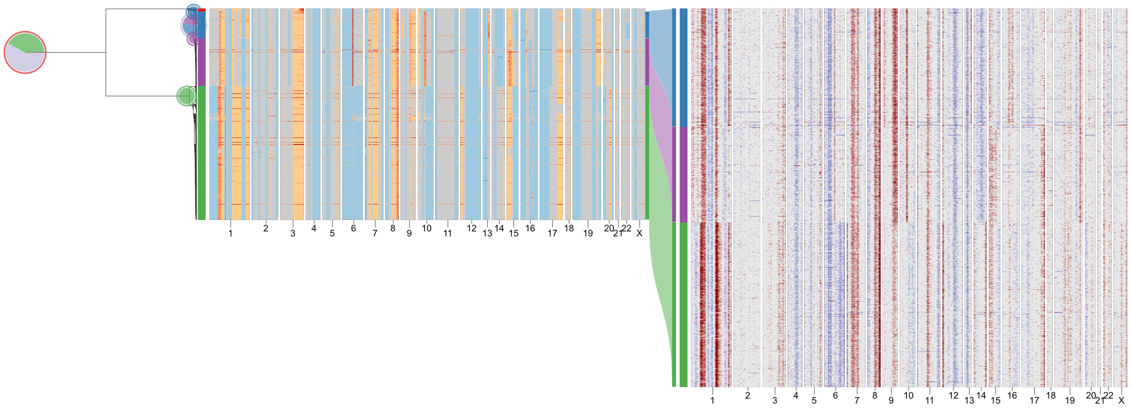

Fig. S43. Total CN model of TreeAlign assigns expression profiles of SA1053BX1XB01603 to phylogeny. Heat maps of copy number profiles from scDNA (left) and InferCNV corrected expression profiles from scRNA (right). The Sankey chart in the middle shows clone assignment from expression profiles to copy number based clones by total CN TreeAlign.

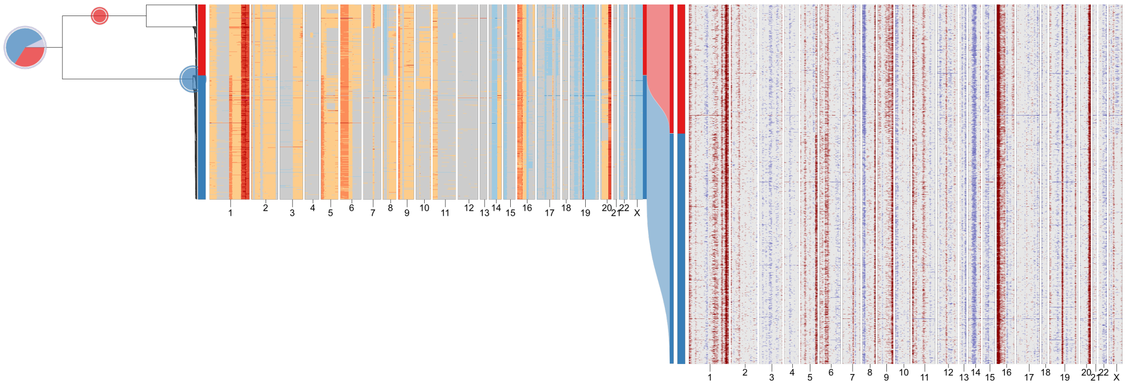

Fig. S44. Total CN model of TreeAlign assigns expression profiles of SA1091AX1XB01790 to phylogeny. Heat maps of copy number profiles from scDNA (left) and InferCNV corrected expression profiles from scRNA (right). The Sankey chart in the middle shows clone assignment from expression profiles to copy number based clones by total CN TreeAlign.

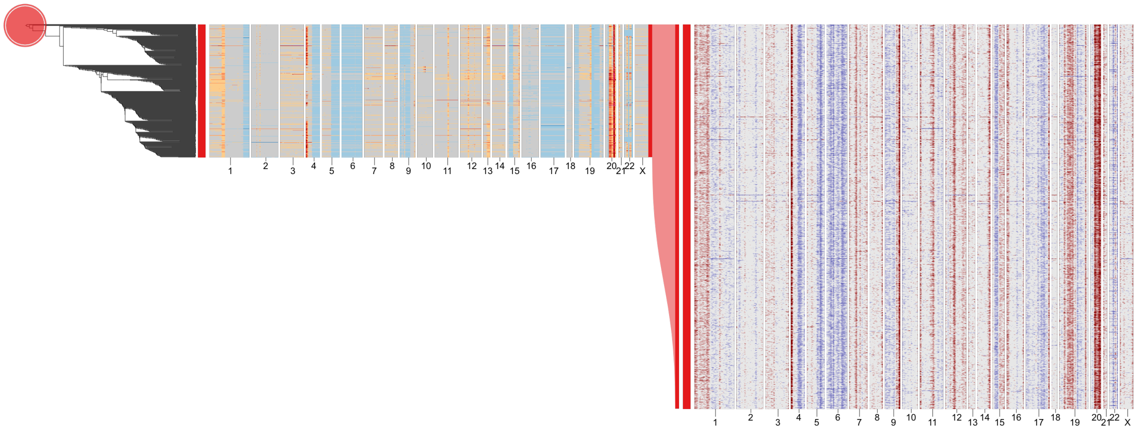

Fig. S45. Total CN model of TreeAlign assigns expression profiles of SA1093CX1XB01917 to phylogeny. Heat maps of copy number profiles from scDNA (left) and InferCNV corrected expression profiles from scRNA (right). The Sankey chart in the middle shows clone assignment from expression profiles to copy number based clones by total CN TreeAlign.

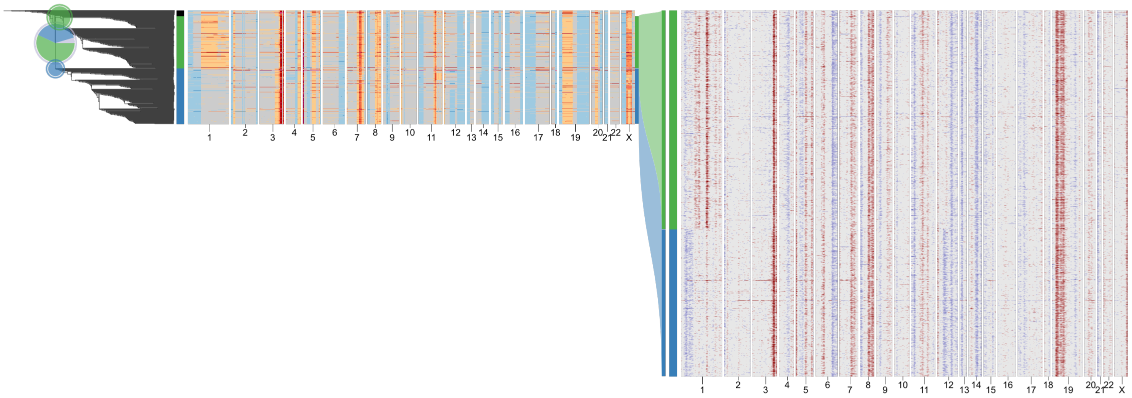

Fig. S46. Total CN model of TreeAlign assigns expression profiles of SA1181AX1XB02700 to phylogeny. Heat maps of copy number profiles from scDNA (left) and InferCNV corrected expression profiles from scRNA (right). The Sankey chart in the middle shows clone assignment from expression profiles to copy number based clones by total CN TreeAlign.

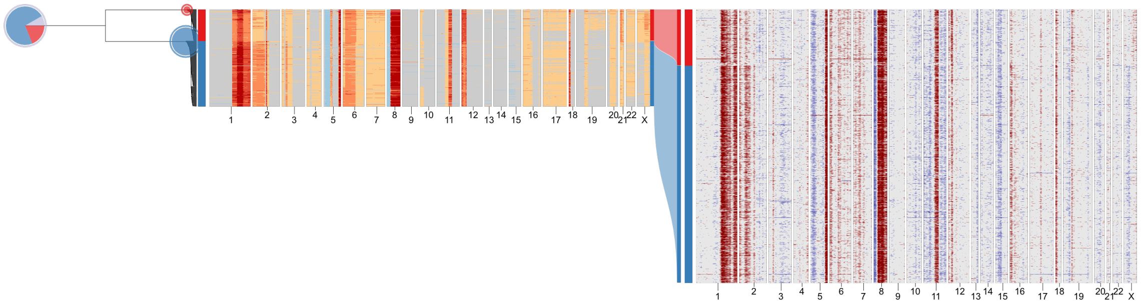

Fig. S47. Total CN model of TreeAlign assigns expression profiles of SA610X3XB03802 to phylogeny. Heat maps of copy number profiles from scDNA (left) and InferCNV corrected expression profiles from scRNA (right). The Sankey chart in the middle shows clone assignment from expression profiles to copy number based clones by total CN TreeAlign.

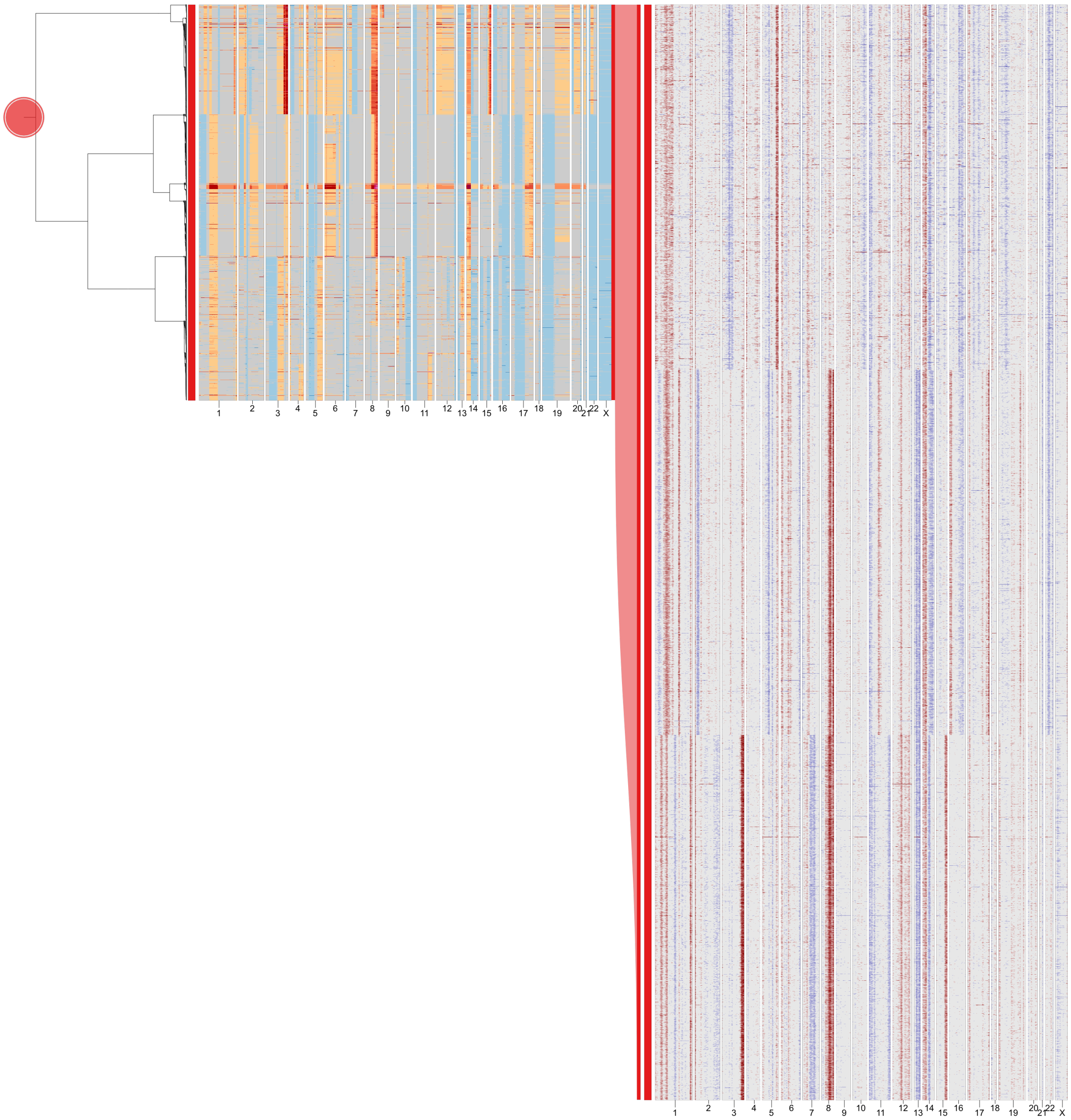

Fig. S48. Total CN model of TreeAlign assigns expression profiles of OV2295 to phylogeny. Heat maps of copy number profiles from scDNA (left) and InferCNV corrected expression profiles from scRNA (right). The Sankey chart in the middle shows clone assignment from expression profiles to copy number based clones by total CN TreeAlign.

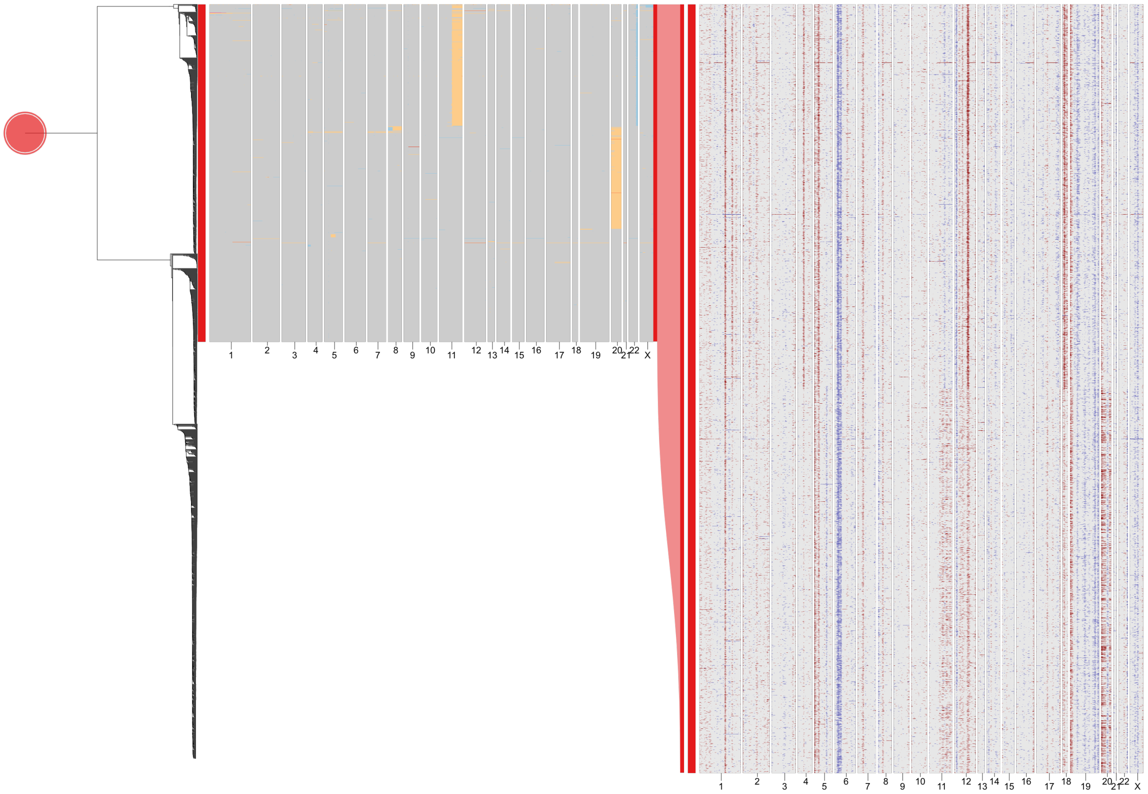

Fig. S49. Total CN model of TreeAlign assigns expression profiles of SA039 to phylogeny. Heat maps of copy number profiles from scDNA (left) and InferCNV corrected expression profiles from scRNA (right). The Sankey chart in the middle shows clone assignment from expression profiles to copy number based clones by total CN TreeAlign.

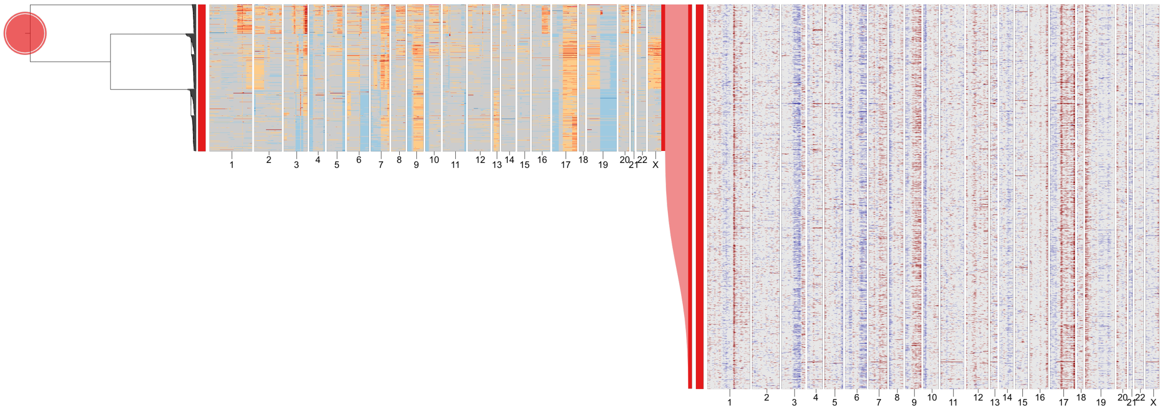

Fig. S50. Total CN model of TreeAlign assigns expression profiles of SA1054 to phylogeny. Heat maps of copy number profiles from scDNA (left) and InferCNV corrected expression profiles from scRNA (right). The Sankey chart in the middle shows clone assignment from expression profiles to copy number based clones by total CN TreeAlign.

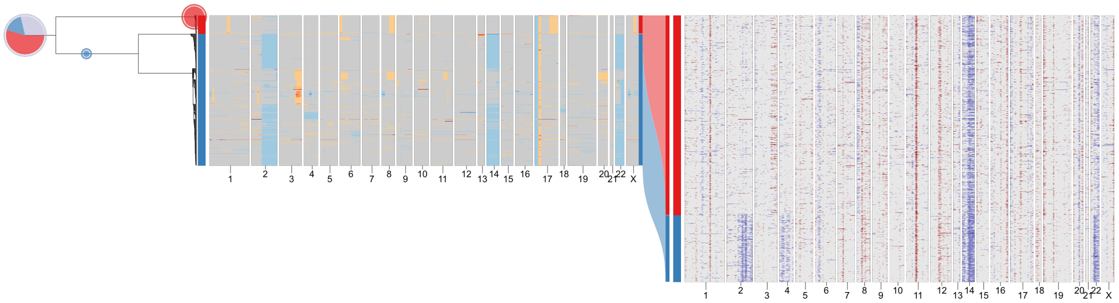

Fig. S51. Total CN model of TreeAlign assigns expression profiles of SA1055 to phylogeny. Heat maps of copy number profiles from scDNA (left) and InferCNV corrected expression profiles from scRNA (right). The Sankey chart in the middle shows clone assignment from expression profiles to copy number based clones by total CN TreeAlign.

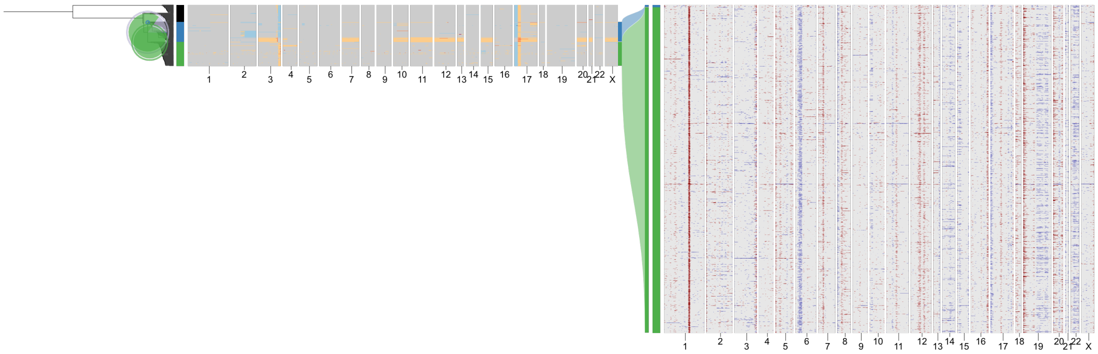

Fig. S52. Total CN model of TreeAlign assigns expression profiles of SA1188 to phylogeny. Heat maps of copy number profiles from scDNA (left) and InferCNV corrected expression profiles from scRNA (right). The Sankey chart in the middle shows clone assignment from expression profiles to copy number based clones by total CN TreeAlign.

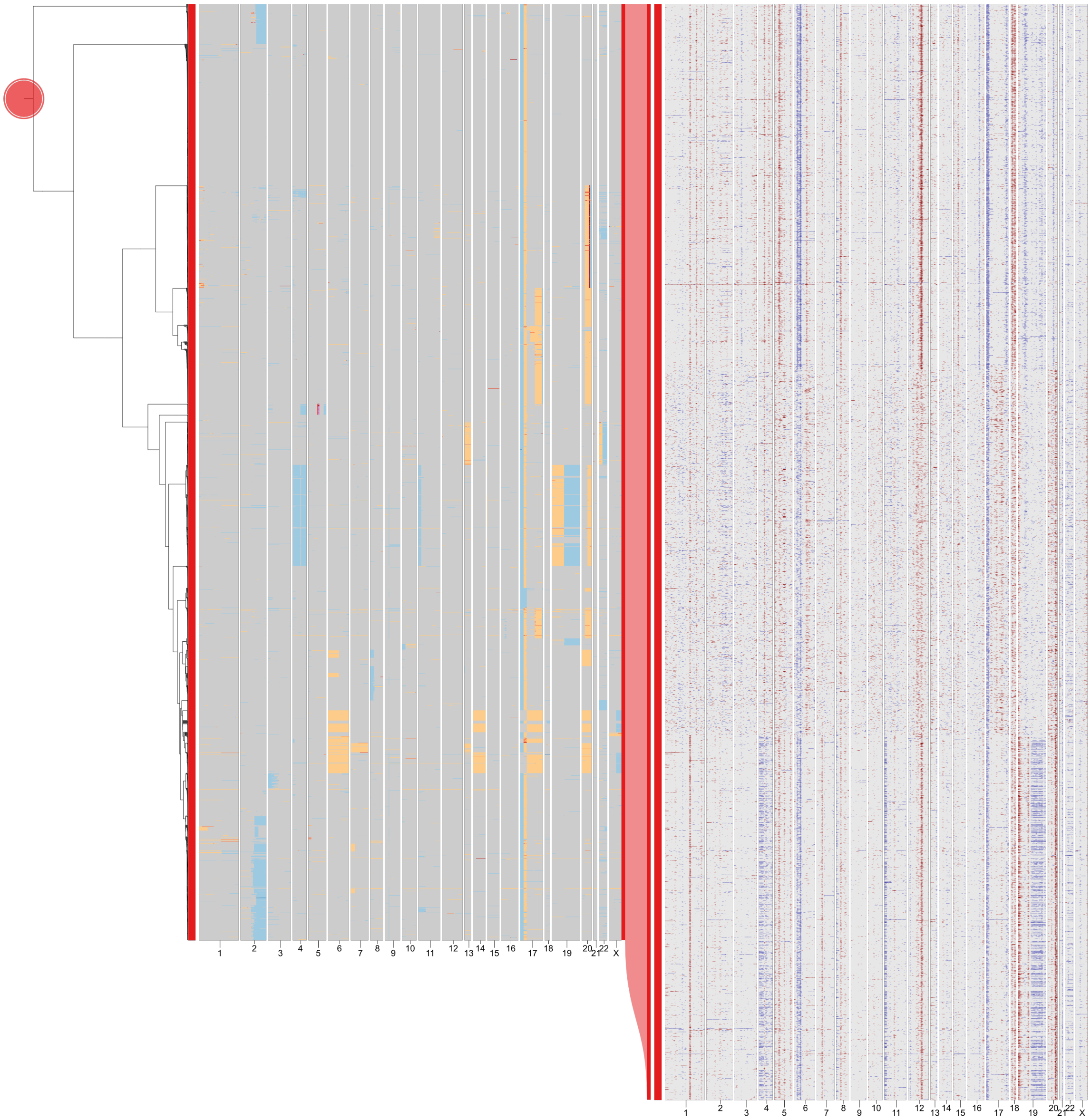

Fig. S53. Total CN model of TreeAlign assigns expression profiles of SA906b to phylogeny. Heat maps of copy number profiles from scDNA (left) and InferCNV corrected expression profiles from scRNA (right). The Sankey chart in the middle shows clone assignment from expression profiles to copy number based clones by total CN TreeAlign.
