# Supplementary material for: Allele-specific transcriptional effects of subclonal copy number alterations enable genotype-phenotype mapping in cancer cells
Source: Nat Commun. 2024 Mar 20;15:2482. doi: 10.1038/s41467-024-46710-0 (PMC10954741; doi:10.1038/s41467-024-46710-0)
Supplement: Supplementary file 3 — Reporting Summary [file 41467_2024_46710_MOESM3_ESM.pdf]

Corresponding author(s): Sohrab Shah
Last updated by author(s): Nov 1, 2023

Reporting Summary

Nature Portfolio wishes to improve the reproducibility of the work that we publish. This form provides structure for consistency and transparency in reporting. For further information on Nature Portfolio policies, see our Editorial Policies and the Editorial Policy Checklist.

Statistics

For all statistical analyses, confirm that the following items are present in the figure legend, table legend, main text, or Methods section.

n/a	Confirmed	
☐	☒	The exact sample size (n) for each experimental group/condition, given as a discrete number and unit of measurement
☒	☐	A statement on whether measurements were taken from distinct samples or whether the same sample was measured repeatedly
☐	☒	The statistical test(s) used AND whether they are one- or two-sided<br>*Only common tests should be described solely by name; describe more complex techniques in the Methods section.*
☒	☐	A description of all covariates tested
☐	☒	A description of any assumptions or corrections, such as tests of normality and adjustment for multiple comparisons
☐	☒	A full description of the statistical parameters including central tendency (e.g. means) or other basic estimates (e.g. regression coefficient) AND variation (e.g. standard deviation) or associated estimates of uncertainty (e.g. confidence intervals)
☐	☒	For null hypothesis testing, the test statistic (e.g. F, t, r) with confidence intervals, effect sizes, degrees of freedom and P value noted<br>*Give P values as exact values whenever suitable.*
☐	☒	For Bayesian analysis, information on the choice of priors and Markov chain Monte Carlo settings
☒	☐	For hierarchical and complex designs, identification of the appropriate level for tests and full reporting of outcomes
☐	☒	Estimates of effect sizes (e.g. Cohen's d, Pearson's r), indicating how they were calculated

Our web collection on statistics for biologists contains articles on many of the points above.

Software and code

Policy information about availability of computer code

Data collection	no software was used
Data analysis	The code for TreeAlign is publicly accessible on a GitHub repo (https://github.com/shahcompbio/TreeAlign). Other algorithms and software used for data analysis include: SIGNALS (https://github.com/shahcompbio/signals), Pyro 1.8.1, CloneAlign v.2.0, InferCNV v.1.3.5, HDBSCAN, Sitka, CellRanger v.3.1.0, Seurat v.4.2, fgsea v.1.24.0

For manuscripts utilizing custom algorithms or software that are central to the research but not yet described in published literature, software must be made available to editors and reviewers. We strongly encourage code deposition in a community repository (e.g. GitHub). See the Nature Portfolio guidelines for submitting code & software for further information.

Data

Policy information about availability of data

All manuscripts must include a data availability statement. This statement should provide the following information, where applicable:

- Accession codes, unique identifiers, or web links for publicly available datasets
- A description of any restrictions on data availability
- For clinical datasets or third party data, please ensure that the statement adheres to our policy

Processed data containing input and output of TreeAlign have been deposited in Zenodo [https://doi.org/10.5281/zenodo.7517412]. Raw scDNA data and scRNA count matrix of the gastric cancer cell line (NCI-N87) can be accessed from SRA (PRJNA498809) [https://www.ncbi.nlm.nih.gov/bioproject/?term=PRJNA498809]

and GEO (GSE142750)[https://www.ncbi.nlm.nih.gov/geo/query/acc.cgi?acc=GSE142750]. Raw scDNA and scRNA data from Funnell et al. are available at [https://ega-archive.org/studies/EGAS00001006343]. Raw scRNA data for patient 022 and patient 081 are available at [https://www.synapse.org/msk_spectrum]. Hallmark gene sets were downloaded from MSigDB [https://www.gsea-msigdb.org/gsea/msigdb/human/genesets.jsp?collection=H]. Source data are provided as a Source Data file.

Research involving human participants, their data, or biological material

Policy information about studies with human participants or human data. See also policy information about sex, gender (identity/presentation), and sexual orientation and race, ethnicity and racism.

Reporting on sex and gender	Data from patient 081 and patient 022 from study cohort MSK SPECTRUM were used in this study. All patients are female with high-grade serous ovarian cancer.
Reporting on race, ethnicity, or other socially relevant groupings	Race, ethnicity, or other socially relevant groupings were not considered as they are irrelevant in this study
Population characteristics	Data from 2 patients: patient 081 and patient 022 from cohort MSK SPECTRUM were used. The study cohort (MSK SPECTRUM) includes 42 women with newly diagnosed, treatment-naive high-grade serous ovarian cancer (HGSOC). Patients between the ages of 39 and 81 at diagnosis (median age: 61 years). 6 out of 42 cases had BRCA1 mutations (14%) and 1 out of 42 cases had a BRCA2 mutation (2%).
Recruitment	All enrolled patients were consented to an institutional biospecimen banking protocol and a protocol to perform targeted panel sequencing (MSK-IMPACT). All analyses were performed per a biospecimen research protocol. All protocols were approved by the Institutional Review Board (IRB) of Memorial Sloan Kettering Cancer Center (MSKCC). Patients were consented following the IRB-approved standard operating procedures for informed consent. Written informed consent was obtained from all patients before conducting any study-related procedures. This study was conducted in accordance with the Declaration of Helsinki and the Good Clinical Practice guidelines (GCP).
Ethics oversight	Institutional Review Board (IRB) at Memorial Sloan Kettering Cancer Center (MSKCC).

Note that full information on the approval of the study protocol must also be provided in the manuscript.

Field-specific reporting

Please select the one below that is the best fit for your research. If you are not sure, read the appropriate sections before making your selection.

☒ Life sciences ☐ Behavioural & social sciences ☐ Ecological, evolutionary & environmental sciences

For a reference copy of the document with all sections, see nature.com/documents/nr-reporting-summary-flat.pdf

Life sciences study design

All studies must disclose on these points even when the disclosure is negative.

Sample size	In this study, we conducted DLP+ to collect scDNA data from 1 patient (patient 022) for validating TreeAlign model. Other data use are all from public sources including data from a gastric cell line NCI-N87 from Andor et al., PDXs of Triple Negative Breast Cancer (TNBC) (n = 3) and HGSC (n = 6), ovarian cancer control cell line (n=1) and 184-hTERT cell lines (n=6) from Funnell et al.
Data exclusions	No exclusions
Replication	N/A. Replication is irrelevant as patient and cell line data were used for model validation/demonstration and we did not make clinical/biological conclusions.
Randomization	N/A. The goal of the study is to build a new computational tool for scDNA and scRNA data integration. Patient and cell line data were used for model validation/demonstration.
Blinding	N/A. There is no group allocation in this study.

Reporting for specific materials, systems and methods

We require information from authors about some types of materials, experimental systems and methods used in many studies. Here, indicate whether each material, system or method listed is relevant to your study. If you are not sure if a list item applies to your research, read the appropriate section before selecting a response.

Materials & experimental systems

n/a	Involved in the study
☒	☐ Antibodies
☒	☐ Eukaryotic cell lines
☒	☐ Palaeontology and archaeology
☒	☐ Animals and other organisms
☒	☐ Clinical data
☒	☐ Dual use research of concern
☒	☐ Plants

Methods

n/a	Involved in the study
☒	☐ ChIP-seq
☒	☐ Flow cytometry
☒	☐ MRI-based neuroimaging

Plants

Seed stocks

Report on the source of all seed stocks or other plant material used. If applicable, state the seed stock centre and catalogue number. If plant specimens were collected from the field, describe the collection location, date and sampling procedures.

Novel plant genotypes

Describe the methods by which all novel plant genotypes were produced. This includes those generated by transgenic approaches, gene editing, chemical/radiation-based mutagenesis and hybridization. For transgenic lines, describe the transformation method, the number of independent lines analyzed and the generation upon which experiments were performed. For gene-edited lines, describe the editor used, the endogenous sequence targeted for editing, the targeting guide RNA sequence (if applicable) and how the editor was applied.

Authentication

Describe any authentication procedures for each seed stock used or novel genotype generated. Describe any experiments used to assess the effect of a mutation and, where applicable, how potential secondary effects (e.g. second site T-DNA insertions, mosiacism, off-target gene editing) were examined.
